# Efficacy, characteristics, behavioural models and behaviour change strategies, of non-workplace interventions specifically targeting sedentary behaviour; a systematic review and meta-analysis of randomised control trials in healthy ambulatory adults

**DOI:** 10.1371/journal.pone.0256828

**Published:** 2021-09-07

**Authors:** Fiona Curran, Catherine Blake, Caitriona Cunningham, Carla Perrotta, Hidde van der Ploeg, James Matthews, Grainne O’Donoghue

**Affiliations:** 1 School of Public Health, Physiotherapy and Sports Science, University College Dublin, Dublin, Ireland; 2 Department of Public and Occupational Health, Amsterdam Public Health Research Institute, Amsterdam UMC, Vrije Universiteit, Amsterdam, Netherlands; University of Maryland School of Medicine, UNITED STATES

## Abstract

**Background:**

Sedentary behaviour (SB) research has grown exponentially but efficacy for interventions to reduce sedentary behaviour is often contaminated by interventions primarily or co-targeting other behaviours and outcomes. The primary aim of this research therefore, was to systematically review the efficacy of interventions specifically targeting sedentary behaviour reduction, as a sole primary outcome, from randomised control trials in healthy ambulatory adults. This research also sought to identify the successful interventions characteristics, behaviour change techniques (BCT’s) and underlying theories, and their relation to intervention effectiveness.

**Methods:**

We followed PRISMA reporting guidelines for this systematic review. Six electronic databases were searched and a grey literature review conducted. Only randomised or cluster randomised controlled trials, from 2000 to 2020, in adult populations with a sole primary outcome of change in sedentary behaviour were included. Data codebooks were developed, data were extracted, and a narrative synthesis and meta-analysis was conducted using mixed methods random effects models.

**Results:**

Of 5589 studies identified, 7 studies met the inclusion criteria. Six studies reported activPAL3 measures of mean daily sitting time, and four reported mean daily standing time, stepping time and number of sedentary breaks. Pooled analysis of weighted mean differences revealed a reduction in mean daily sitting time of -32.4mins CI (-50.3, -14.4), an increase in mean daily standing time of 31.75mins CI (13.7, 49.8), and mean daily stepping time of 9.5mins CI (2.8, 16.3), and an increase in rate of sedentary breaks per day of 3.6 (CI 1.6, 5.6). BCTs used exclusively in two of the three most effective interventions are ‘feedback on behaviour’ and ‘goal setting behaviour’ whilst all three most effective interventions included ‘instruction on how to perform the behaviour’ and ‘adding objects to the environment’, BCTs which were also used in less effective interventions.

**Conclusions:**

Although limited by small sample sizes and short follow up periods, this review suggests that interventions specifically designed to change sedentary behaviour, reduce overall daily sitting time by half an hour, with an equivalent increase in standing time, in the short to medium term. Effective characteristics and behaviour change strategies are identified for future development of high quality interventions targeting change in sedentary behaviour.

**Prospero registration:**

PROSPERO 2020 CRD42020172457 Available from: https://www.crd.york.ac.uk/prospero/display_record.php?ID=CRD42020172457.

## Introduction

Sedentary behaviour (SB), defined as any waking behaviour characterized by an energy expenditure ≤1.5 metabolic equivalents (METs), while in a sitting, reclining or lying posture [1], has been identified as an independent risk factor for morbidity and mortality [2–5]. A number of recent national and international advisories highlight the potential health risk posed by sedentary behaviours, and encourage the development of public health strategies and guidelines to reduce these behaviours across all age groups and domains [6–9]. Sedentary behaviour is distinct from physical inactivity, which is defined as ‘an insufficient physical activity level to meet present physical activity recommendations’ [1]. Thus a person can be physically inactive but not engage in prolonged sedentary behaviour or vice versa, and increased levels of physical activity do not necessarily equate with reductions in sedentary behaviour. Guidelines regarding health enhancing physical activity (HEPA) are widely published and adopted in public health strategies and policies [10–13]. However, the development of strategies and policy to interrupt and reduce sedentary behaviour is hindered by the misconception that physical inactivity is synonymous with sedentary behaviour- [14, 15], by the lack of cross-domain and non-workplace based interventions, and by the complexity of identifying the ‘active ingredients’ of effective interventions to translate into practice [16]. The updated World Health Organisation (WHO) guidelines strongly recommend that adults limit sedentary time, replacing it with any intensity of physical activity (PA), and offset high levels of sedentary behaviour with increases in moderate to vigorous PA [17]. Other recent PA guidelines include information and resources on sedentary behaviour, and non-specific recommendations to minimise overall sedentary behaviour and prolonged sitting bouts [9–13].

Although sedentary behaviour research has grown exponentially in the last decade, the main focus of much of the intervention research is on physical activity, with change in sedentary behaviour as a secondary focus only [18–24]. Moreover, sedentary behaviour research has most frequently been focused on workplace interventions with a paucity of cross-domain or non- occupational interventions [23, 25–29]. These factors contribute to the difficulty identifying effective sedentary behaviour interventions, and to the inability to translate effective interventions into practice across all domains of living and particularly into community or domestic domains.

Of the systematic reviews and meta-analyses synthesising sedentary behaviour interventions that have been published over the past decade [30–40], most report interventions which primarily target other behaviours e.g. physical activity or lifestyle [31, 35, 36, 38] and sedentary behaviour is a secondary or combined outcome; focus on, or include children in their analyses [33, 37, 39]; or focus on changes in workplace sitting time [34, 40]. In a review of interventions to reduce sitting time, sixty-three percent of studies focused solely on physical activity, while only twenty-one percent (eight studies) focused solely on sedentary behaviour reduction, and of those, six are workplace based [36]. Furthermore, the majority of reviews include non-randomised or non-controlled clinical trials which do not elucidate the highest level evidence [30–32, 34, 36, 39, 40]. One of the seminal systematic reviews published in 2014 [32] which compared interventions targeting physical activity, or co- targeting physical activity and sedentary behaviour, with interventions solely targeting sedentary behaviour, concluded that sedentary behaviour reduction was best achieved by interventions which specifically and solely targeted sedentary behaviour reduction in their design and implementation. This finding is replicated by Gardner et al [36] and more recently in a review of interventions using self-monitoring to interrupt sedentary behaviour [30]. However, many studies continue to implement interventions which co-target sedentary behaviour change with other primary outcomes, e.g. PA or diet. The purpose of this review is to find highest level evidence for a community based sedentary behaviour intervention, by including only RCTs which have a strong non-workplace component, and whose sole primary outcome is change in sedentary behaviour.

Behaviour change techniques (BCTs) are the observable, replicable and irreducible components of behaviour change or the ‘active ingredients’ in behaviour change interventions [41]. Identifying these potentially active ingredients and linking them with the mechanisms of action, or processes through which behaviour change occurs, is necessary to effectively elicit the behavioural change desired [42]. Thus, process evaluations of sedentary behaviour interventions are fundamental to understanding intervention content, mechanisms of action, implementation and delivery approaches, and contexts, and critically, their association with effectiveness. Furthermore, any BCTs identified must be stringently linked to the behaviour change being investigated and interventions must also be underpinned by theory which is explicitly linked to mechanisms of action and BCTs [43, 44]. Whilst one review to date has led the way in process evaluation of sedentary behaviour, it also highlights the need for research focussing solely on sedentary behaviour, for the inclusion of process evaluation, and for data extraction around BCTs to be specifically focussed on sedentary behaviour [36]

Hence, identifying the components of successful interventions that focus solely on reducing sedentary behaviour is necessary to inform the development of future interventions, to effect clinically significant change. Effective sedentary behaviour focused interventions will contribute towards the development of clear clinical and public health sedentary reduction guidelines and strategies, and thereby, have the potential to alleviate associated morbidity and mortality.

The overarching objective of this review therefore, is to synthesise the highest level evidence for efficacy of interventions that target sedentary behaviour reduction as a sole primary outcome in healthy ambulatory adults. Secondary objectives are 1) to explore the core components of these interventions, specifically in relation to intervention characteristics (i.e. method and context of delivery, by whom, with what intensity and for how long) and behaviour change theory and techniques; 2) to examine to what extent intervention effectiveness varies across studies depending on their theoretical basis, BCTs, and intervention features.

## Methods

### Registration

This systematic review is reported in accordance with the Preferred Reporting Items for Systematic Reviews and Meta-Analyses (PRISMA) statement [45]. The review was prospectively registered on April 28, 2020 (PROSPERO 2020: CRD42020172457) with the International Prospective Register of Systematic Reviews (PROSPERO).

### Search strategy

Six electronic databases (PubMed, EMBASE, Cochrane Central Register of Controlled Trials (CENTRAL), Cumulative Index to Nursing and Allied Health Literature (CINAHL), PsycINFO and SPORTDiscus) were searched. The search strategy was constructed in collaboration with a research librarian, around the PICOS tool; (P) Population: sedentary adults, (I) Intervention: any intervention specifically targeting sedentary behaviour as a sole primary outcome, (C) Comparator: usual behaviour, wait-list control, placebo, (O) Outcomes; time spent sedentary and (S) Study type: randomised controlled trials. A complete list of the search terms is available in the additional materials section (S1 Table). In addition to the databases, the reference lists of included articles were hand searched for articles that met the inclusion criteria.

#### Eligibility criteria

Since the study of sedentary time is a relatively new area with a rapid growth in recent years the search was limited to the last 20 years. Randomised controlled trials (RCTs) published in scientific peer reviewed papers, written in English (due to language limitations of the research team), between January 2000–December 2020 were included (conference abstracts, reports and theses were excluded). The population, “adult” was defined by the individual study in the range 16–69 years. One study may define adults as over 18 while another may define it as over 16. Studies including children or adolescents, were excluded. Studies whose target population was older adults (>65 years), or people with a diagnosed pathology (e.g. type 2 diabetes) were excluded. Only interventions specifically designed to change sedentary behaviour were included. Thus, if sedentary time was a combined primary outcome (e.g. sedentary behaviour and physical activity targeted), or a secondary outcome of a study designed primarily to target a different behaviour (e.g. physical activity), it was excluded. Originally, interventions across all domains were included, but recent systematic reviews have focussed on workplace sitting time [35, 46, 47], and workplace interventions may not transfer to community or leisure time. Thus, workplace interventions designed to change workplace sedentary behaviour alone were excluded, but cross domain interventions which included workplace and leisure time or domestic components were included if the objective was to change overall sedentary behaviour. However, all eligible non-workplace interventions were included even if the total day was not addressed, so that sedentary behaviour in this domain can be targeted in future studies. In terms of sedentary behaviour outcome measures, the following were acceptable: change in total sedentary time (in sitting or reclining position) and/or change in sedentary bouts (frequency/duration of breaks). Both self-reported or device based (accelerometry and inclinometry) estimates of sedentary behaviour were included. In addition to sedentary behaviour, other outcomes of interest included anthropometry (body weight (BW), BMI, percentage body fat (%BF), waist circumference (WC)), cardiorespiratory fitness as measured by maximal oxygen uptake (V0_2 max_), and risk factors associated with the metabolic syndrome (systolic and diastolic blood pressure (BP), total high-density lipoprotein cholesterol (HDL), triglycerides (TG), fasting blood glucose (FBG) and glycated haemoglobin (HbA1c)).

### Data collection and extraction

All studies were imported into EndNote (Version X9) and de-duplicated. Two authors (FC, GO’D) independently screened titles and subsequently abstracts for potential inclusion and following review for accuracy, full text was retrieved and independently screened for potentially eligible studies. Any disagreement over the eligibility of particular studies was resolved through discussion with a third reviewer (CC) and consensus reached.

Microsoft Excel was used to develop comprehensive electronic codebooks by two authors with feedback from a third author, for study characteristics, demographics, primary and secondary outcomes (FC, GO’D, CP), BCTs (FC, JM, GO’D), quality assessment (FC, CC, GO’D) and intervention characteristics were coded according to the template for intervention description and replication (TIDieR) framework [48] (FC, CC, JM). Thus the extraction and coding process, for each of the variables coded, was standardised. The same authors subsequently independently extracted data into these codebooks or quality checked the data.

The BCT Taxonomy v1, “a cross-domain, hierarchically structured taxonomy of 93 distinct BCTs with labels, definitions and examples” [41] was used to code BCTs. Two BCT V1 taxonomy trained authors (FC, JM) independently extracted and coded the BCT data from each study, and any disagreement was resolved by discussion. Final agreement was reached by discussion with a third BCT V1 taxonomy trained author (GO’D). Intervention and control conditions were coded separately and as directed in the BCT taxonomy, BCTs were coded only if clearly linked to the target behaviour change (i.e. sedentary behaviour).

#### Data synthesis and quality assessment

Firstly, the included trials are qualitatively described. The narrative synthesis is structured around the characteristics of the studies, including populations, primary and secondary outcomes, the theory, characteristics, and application of the interventions using the TIDieR framework [48] as an extension of Item five of the CONSORT statement [49]. Behaviour change techniques are identified according to the BCT taxonomy V1, synthesised and discussed in relation to the effectiveness of the interventions. Finally a quality assessment of the included studies, using the Cochrane risk of bias assessment tool is reported [50].

### Data analysis strategy

Quantitative analysis was conducted in Stata (version 15). Continuous outcome measures were expressed as mean or rate with SD and then converted to standard units (standardised mean difference (SMD)). If the standard deviation (SD) difference was missing, it was calculated using the SD formula of the difference between two means:$\sqrt[{2}]{\frac{{\text{s}}_{1}{}^{2}}{{\text{n}}_{1}}+\frac{{\text{s}}_{2}{}^{2}}{{\text{n}}_{2}}}$. Due to the heterogeneity of the interventions and the low number of studies, it was not possible to pool studies according to intervention type. Instead, studies were pooled according to comparable outcome measure. Pooled effects were estimated for daily sitting time, standing time, stepping time and number of sedentary breaks per day. Pooled effects were based on intervention effects (mean between-groups difference) for the end-of-intervention final follow-up endpoint and estimated from random effects pairwise meta-analysis using Der Simonian and Laird Model, with the *I*^2^ statistic quantifying heterogeneity. Significance was set at p<0.05 (two tailed). Findings from the meta-analysis are presented using forest plots. Only one study in the review had multiple arms, and our intention was to split the control to include half in each meta-analysis as per the Cochrane handbook [50], but the study did not have variables matched for the meta-analysis, and was therefore not included. Planned a priori subgroup analyses for BMI categories and domains of living was not possible due to lack of data and exclusion of workplace interventions.

## Results

### Literature selection

**Fig 1:**
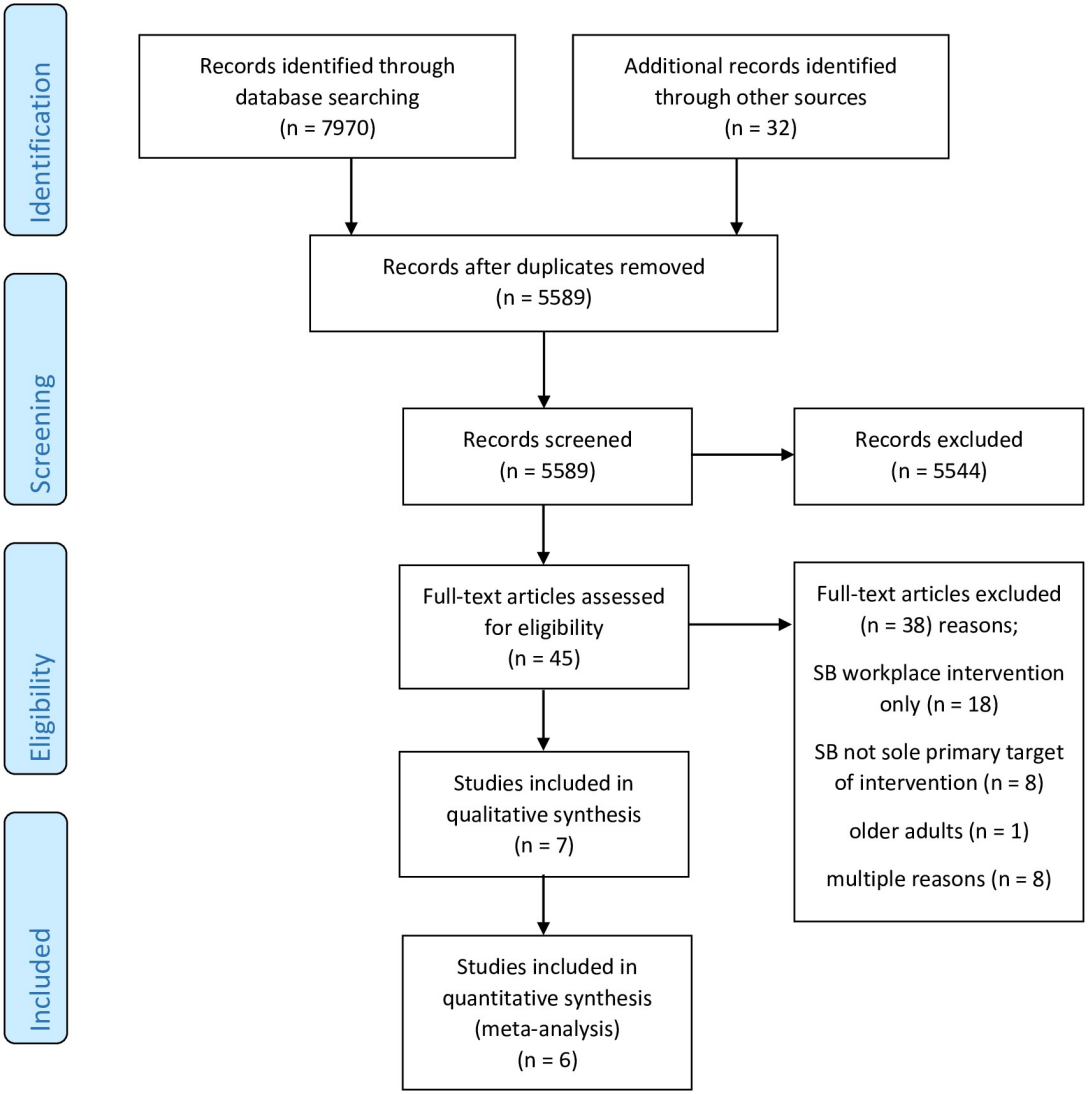
PRISMA flow diagram.

A total of 5589 studies were initially identified. Following review by title and abstract, 45 studies progressed to full manuscript review. Of these, 38 were excluded as they did not fulfil our inclusion criteria. Details of full text exclusions are available in the additional materials section (S2 Table). The remaining 7 studies were included in this review. The detailed process is illustrated in Fig 1.

### Study characteristics

The characteristics of the included studies are presented in Table 1. Only three studies published a CONSORT [49] flow diagram [51–53], two of which also published a CONSORT checklist and one study reported that it based its intervention design on the guidelines.

**Table 1:** Study characteristics.

Study year	Sample size	Country	Population	RCT Study Design	Age Mean, (SD)	Female n, (%)	Intervention	Final Follow-up (weeks)	SB measurement	BMI	Working %
										Mean, (SD) (overweight or obese %)	
Aadahl 2014	166	Denmark	Age 18–69 years; 3.5 hrs/day LT SB; max vigorous PA 8 hrs/week	2 arm parallel	52, (14.1)	95, (57.2)	motivational counselling to reduce sitting	26	activPAL	27.3, (5)	52%
Arrogi 2019	58	Belgium	Age 18–55 years; desk bound job & /or sedentary LT;	2 arm parallel	36.2, (9.9)	30, (51.7)	smartphone app (stapp)	2	activPAL	-, -	98%
			au-fait with a smartphone								
Biddle 2015	187	England	Age 18–40 years;	2 arm parallel	32.8, (5.6)	130, (69.5)	structured education group workshop focused on sitting reduction, and self-monitoring device.	52	activPAL	34.6, (4.9) (84.5)	-
			BMI obese (>30kg/m2 or >27.5kg/m2 for sth Asians) or overweight (>25kg/m2 or >23kg/m2 for South Asians) & one or more additional risk factor for diabetes								
Ellingson 2016	28	USA	full-time students;	pilot 2 arm parallel	20.1, (1.5)	14, (50.0)	sedentary feedback via activPAL	10	activPAL	22.8, (4.6)	-
			age 18–26 years.;								
			No mobility limitations;								
			> 3hrs/day self report LT SB.								
Judice 2015	20	Portugal	Age 18–65 years;	pilot crossover	50.4, (11.5)	5, (25.0)	Cross-domain—computer prompts and goal setting	2	activPAL	32.6, (5.5) (80.0)	100%
			employed in academic/admin;								
			>7hrs/day computer work.								
			BMI >25; healthy								
			physically inactive								
Kitigawa 2020	48	Japan	Age 20–49 years.;	pilot 3 arm parallel	37.9, (4.4)	48, (100.0)	smartphone app with self feedback or tailored feedback	2	activPAL & UP24	21.5, (4.1)	0%
			housewife (no paid work & dedicated to housework);								
			has child below primary school; healthy;								
Nishimuru 2019	26	Japan	Age 30–69 years;	pilot 2 arm parallel	51, (9.5)	19, (73.1)	immediate vibrotactile feedback via activPAL; instruction to stand for at least 1 mins	8	activPAL	21.2, (2.6)	77%
			self report SB time >8hrs/d								

LT = leisure time; SB = sedentary behaviour; PA = Physical Activity

Of the seven included studies, four were conducted in Europe [51–54], two in Asia [55, 56] and the remaining one in the United States (US) [57]. As per inclusion criteria, all seven studies were RCTs, five were two arm parallel trials [52–55, 57], one was a three-arm trial [56] and one was a cross-over trial [51]. Only two studies [52, 53] included > 150 participants and sample sizes in the remaining five studies were small (between 10–58 participants).

In total 533 adults, ranging in age from 18–69 years, with a mean of 39.1 (SD 7.8) years were investigated, although there was considerable variability in the age profiles across the studies. In terms of gender, there were more women (n = 341) than men (n = 192) involved in the studies.

Mean BMI was 29.2 (SD 4.7) across the 6 studies in which it was reported (n = 475), [51–53, 55–57]. Three studies reported a healthy mean BMI (<25) (n = 107) [55–57], while one reported mean BMI as overweight (27.5kg/m^2^) [52]. Two studies [51, 53], specifically targeted populations with overweight or obesity, thus of the total population, 69.9% (n = 387) were living with either overweight or obesity (BMI > 25). Overall employment across the seven studies was 61.7% (n = 329), but one study targeted housewives with no paid employment [51] and another targeted full-time students [57], while two studies targeted employees [51, 54].

Intervention duration was extremely varied, ranging from one week in three of the studies [51, 52, 56] to fifty-two weeks [53]. The remaining three study interventions lasted five [57], eight [55], and twenty-six [52] weeks respectively. Study duration and final follow-up also ranged from two to fifty-two weeks.

### Measurement of sedentary behaviour

All seven studies used device based measures of sedentary behaviour although the measures reported were not standardised across the studies. Table 2 provides details. One study used the UP24 Jawbone accelerometer, [56] while the activPAL accelerometer was used by all six others [51–55, 57] and four reported using daily logs to verify the data [51–54]. An ACTigraph GX3 accelerometer was also used in three studies [51, 53, 57] but the outcomes reported varied across these studies. Self-reported measures of sedentary behaviour were also used in three studies, physical activity scale (PAS) [52], international physical activity questionnaire (IPAQ) [53, 57], sedentary behaviour questionnaire (SBQ) [57].

**Table 2:** Sedentary behaviour outcome measures as reported.

Reference (Outcome Measurement)		Intervention Group						Control Group							Intervention Vs Control			
		Mean Pre	SD or CI	Mean post	SD or CI	Change in Mean	SD or CI	Mean pre	SD or CI	Mean post	SD or CI	Change in Mean	SD or CI	Mean difference	Lower 95% CI	Upper 95% CI	P-value	adjusted p value
**Aadahl 2014**																		
activPAL	Sitting time (hrs/day)	9.30	1.80	9.00	1.70	-0.27	1.70	9.80	1.80	9.90	1.80	0.06	1.70	-0.32	-0.87	0.24	0.260	0.310
	Standing time (hrs/day)	4.20	1.10	4.40	1.30	0.21	1.00	4.10	1.20	9.10	1.80	-0.22	1.10	0.44	0.80	0.80	0.020	0.020
	Stepping time (hrs/day)	1.80	0.60	1.90	0.60	0.10	0.50	1.70	0.70	1.70	0.70	-0.04	0.60	0.15	-0.44	0.33	0.110	0.130
	Number of Breaks (n/day)	59.90	15.00	60.30	15.00	0.50	14.80	59.20	19.00	59.70	18.00	0.40	12.20	-0.74	-5.80	4.40	0.770	0.690
	Non-sleep wear time (hrs/day)	15.20	1.30	15.30	0.90	0.04	1.00	15.60	0.90	15.40	1.10	-0.21	0.90	0.27	-0.05	0.60	0.090	0.090
Self Report (PAS)	Leisure Sitting time (hrs/day)	5.30	1.80	4.40	1.70	-0.93	1.60	5.00	1.70	4.90	2.20	-0.03	1.70	-0.81	-1.36	-0.27	0.004	0.004
	Work Sitting time (hrs/day)	4.40	2.40	4.00	2.40	-0.41	1.30	4.40	2.40	4.30	2.40	-0.05	1.20	-0.47	-1.06	0.12	0.120	0.110
	Vigorous PA (hrs/week)	1.30	1.60	1.30	2.20	0.07	1.50	0.80	1.40	0.80	2.10	0.05	2.00	0.29	-0.20	0.78	0.250	0.230
**Arrogi 2019**																		
activPAL on weekdays	Total Sitting time (mins/day)	633.90	81.90	593.40	111.5			658.40	74.40	658.10	66.00			-40.10	-76.70	-3.50	< .05	
	Total Standing time (mins/day)	232.70	59.90	270.20	95.60			208.70	56.20	210.80	51.60			35.40	2.30	68.50	< .05	
	Stepping time (mins/day)	93.60	34.30	96.60	34.80			93.00	24.20	91.30	25.00			4.70	-7.70	17.20		
	Total Sitting time (% daily waking hrs)	66.00	8.50	61.80	11.60			68.60	7.70	68.50	6.90			-4.20	-8.00	-4.00	< .05	
	Total Standing time (% daily waking hrs)	24.20	6.20	28.10	10.00			21.70	5.90	22.00	5.40			3.70	0.20	7.10	< .05	
	Total Stepping (% daily waking hrs)	9.70	3.60	10.10	3.60			9.70	2.50	9.50	2.60			0.50	0.80	1.80		
	number of prolonged sitting bouts (n/day)	6.20	1.60	3.40	2.00			6.70	1.80	6.30	1.90			2.30	-3.30	-1.30	< .0001	
	average duration bout (mins/day)	59.20	14.60	46.20	17.00			56.00	8.30	56.00	7.90			-13.00	-21.50	-4.40	< .001	
	Total duration bouts (mins/day)	363.60	107.90	178.80	117.50			372.10	119.00	355.40	120.20			-168.00	-224.5	-111.50	< .0001	
	Number of sed. Breaks (mins/day)	47.30	11.10	53.90	11.00			54.60	14.90	55.40	16.30			5.70	1.00	10.40	< .05	
	Non-sleep wear time (hrs/day)																	
	Daily step count	8103	2592	8319	3309			7838	2143	7809	2445			244	-859	1347		
**Arrogi 2019**																		
activPAL on weekend days	Total Sitting time (mins/day)	552.60	144.80	515.80	101.00			569.70	97.80	566.80	108.10			-33.90	-101.7	33.80		
	Total Standing time (mins/day)	295.10	121.00	334.10	91.20			269.20	74.90	269.70	96.40			38.50	-17.70	94.70		
	Stepping time (mins/day)	112.40	44.90	110.30	47.20			121.30	43.30	123.70	39.60			-4.60	-31.60	22.50		
	Total Sitting time (% daily waking hrs)	57.60	15.10	53.70	10.50			59.30	10.20	59.00	11.30			-3.50	-10.60	3.50		
	Total Standing time (% daily waking hrs)	30.70	12.60	34.80	9.50			28.10	7.80	28.10	10.00			4.00	-1.80	9.90		
	Total Stepping (% daily waking hrs)	11.70	4.70	11.50	4.90			12.60	4.50	12.90	4.10			-0.50	-3.30	2.30		
	number of prolonged sitting bouts (n/day)	4.90	2.40	3.00	2.10			5.20	1.50	5.20	1.50			-1.90	-3.30	-0.50	< .001	
	average duration bout (mins/day)							60.70	15.60	61.10	13.50			-13.50	-25.60	-1.40	< .05	
	Total duration bouts (mins/day)	291.60	157.00	154.60	112.20			303.90	95.10	317.90	109.20			-151.00	-231.7	-70.20	< .0001	
	Number of sed. Breaks (n/day)	52.60	20.20	52.50	15.30			57.40	13.00	57.20	15.30			0.20	-8.30	8.60		
	Daily step count	8976	3696	8769	4415			9881	4447	9679	4178			-5.80	-2548	2536		
Biddle 2015** reports change in mean not pre and post																		
activPAL	average sedentary time (hrs/day)	8.91	(8.59, 9.24)	0.64	(0.13, 1.16)			9.02	(8.73, 9.3)	0.58	(0.06, 1.09)			-0.12	-0.99	0.76		
	total sedentary to upright movements (n/day)	53.40	(50.6, 56.1)	7.96	(3.29, 12.6)			51.90	(49.9, 53.9)	5.63	(0.5, 10.76)			-0.19	-6.99	6.61		
ActiGraphGT3x	average sedentary time (hrs/day)	10.83	(10.50, 11.17)	-0.29	(-0.75, 0.17)			11.01	(10.76, 11.26)	-0.23	(-0.6, 0.14)			0.01	-0.49	0.52		
	average number breaks (n/day)	694.70	(657.2, 732.2)	-1.92	(-42.8, 39.0)			672.30	(639.9, 704.7)	9.56	(-39.9, 59.0)			-2.96	-73.00	67.00		
self report IPAQ	Average sitting time (hrs/day)	8.53	(6.38, 10.68)	-3.45	(-6.76, -0.14)			7.13	(6.44, 7.82)	0.84	(-2.45, 4.14)			-1.61	-5.03	1.82	0.353	
**Ellingson 2016**																		
activPAL	Total sedentary time (mins/day)	600.6	78.2	568.7	67.9			610.1	61.4	579.9	59.1							
	prolonged bouts>30 (mins/day)	409.7	77.2	328.8	116.9	*		404.8	101.5	365.1	79.9							
	short bouts<30 (mins/day)	190.9	48.5	239.9	101.1			205.3	70.1	214.8	61.5							
Self report (SBQ)	Total sedentary time (mins/day)	409.2	168.5	372.3	177.5	*		392.5	168.4	326.7	184.3	*						
ActiGraphGT3x	LPA (mins/day)	175.6	49.4	175.3	44.1			205.6	50.3	223.9	73.1							
	MPA (mins/day)	47.1	16.4	47.9	17.5			42.9	13.5	42.7	11.1							
	VPA (mins/day)	26.7	15.1	27.4	12.4			25.4	12.5	24.7	17.8							
Self report (IPAQ)	MPA (mins/day)	79.3	57.2	69.8	46.6			96.9	93.5	91.9	112.0							
	VPA (mins/day)	9.7	33.5	16.7	33.5			41.9	9.5	32.3	9.8							
**Judice 2015**																		
activPAL	Total Sitting time (hrs/day)			9.55	1.80					11.40	1.48			−1.85	1.25		0.001	
	Total Standing time (hrs/day)			5.16	1.82					4.39	1.40			0.77	0.99		0.04	
	Stepping time (hrs/day)			2.33	0.37					1.24	0.29			1.09	0.41		<0.001	
	Sit to Stand transitions (n/day)			56.90	9.06					53.6	11.00			3.28	7.84		0.22	
	Step count (n/day)			12076	1934					5712	1335			6363	1953		<0.001	
	Sit bouts < 5 mins (n/day)			31.20	8.74					26.40	10.80			4.83	9.68		0.149	
	Sit bouts 5-9mins (n/day)			9.6	2.67					7.92	2.46			1.68	2.79		0.088	
	Sit bouts 10–19 mins (n/day)			9.58	3.07					7.62	1.00			1.96	3.00		0.069	
	Sit bouts20-29 mins (n/day)			4.31	1.55					3.81	1.24			0.50	1.50		0.320	
	Sit bouts 30–59 mins (n/day)			4.09	1.86					3.95	1.03			0.13	1.78		0.805	
	Sit bouts > 60 mins (n/day)			3.13	1.46					3.33	1.36			-0.21	1.03		0.542	
	Stand bouts < 5 mins (n/day)			491.00	88.50					365.00	78.50			125.00	104.00		0.004	
	Stand bouts 5–9 mins (n/day)			5.56	2.92					5.55	2.44			0.01	2.51		0.995	
**Judice 2015 Continued**																		
ActiGraphGT3x	Stand bouts 10–19 mins (n/day)			1.29	0.84					1.49	1.37			-0.21	0.89		0.480	
	Stand bouts20-29 mins (n/day)			1.70	2.12					2.10	3.96			-0.40	3.78		<0.001	
	Stepping bouts < 5 mins (n/day)			19.10	6.67					3.00	2.71			16.10	6.95		<0.001	
	Stepping bouts 5–9 mins (n/day)			2.94	1.03					0.46	0.42			2.48	1.07		0.085	
	Actigraph breaks (n/day)			506.00	106.00					477.00	128.00			29.10	7.50		0.745	
**Kitigawa 2020**																		
	Tailored Feedback Group																	
Accelerometer (UP24-Jawbone)	longest bout sitting time (mins/day)	96.7	28.30	81.70	31.50			88.40	17.10	79.30	17.30							
	Total PA	65.10	27.50	66.90	29.00			62.20	22.40	60.80	18.20							
	Steps (n/day)	7538	3145	7613	2692			7324	2646	7107	2233							
	Self-Feedback Group																	
	longest bout sitting time (mins/day)	76.50	11.4	75.30	20.20			0.01	0.41	0.20	0.27							
	Total PA	72.50	23.60	67.00	18.40			0.32	0.15	0.23	0.26							
	Steps (n/day)	8483	2815	7822	2229			0.37	0.14	0.13	0.31							
**Nishimuru 2019**																		
activPAL	Sedentary time (mins/9hr)			339	64					345	76	17.5	(-32.4,-2.5)					
	Sit to Stand transitions (n/9hr)	46.00	19.00	46.00	17.00	0.60	(-3.4, 4.5)	40.00	11.00	39.00	6.00	-0.40	(-4.3, 3.6)					
	Sit bouts > 30 mins (n/9hr)	142.00	84.00	121.00	69.00	-20.5	(-42.4, 1.3)	151.00	70.00	136.00	52.00	-0.40	(-36.7, 7.0)					
	Standing time (mins/9hr)	137.00	55.00	144.00	53.00	7.50	(-5.3, 20.2)	117.00	48.00	133.00	47.00	15.90	(3.1, 28.7)					
	Stepping time (mins/9hr)	51.00	11.00	55.00	10.00	3.90	(-3.6, 11.5)	59.00	18.00	65.00	17.00	5.90	(-1.7, 13.5)					

^**Abbreviations**; standard deviation (SD); Confidence Interval (CI); hours (hrs); Number (n); Physical Activity Scale (PAS); Physical Activity (PA); Minutes (mins); International Physical Activity Questionnaire (IPAQ); Sedentary Behaviour Questionnaire (SBQ); Light Physical Activity (LPA); Moderate Physical Activity (MPA); Vigorous Physical Activity (VPA)^

In terms of device based sedentary behaviour variables recorded, total daily sitting time was measured in five studies, [51–54, 57] while one study limited the measurement to nine hours per day to ‘limit skin irritation’ [55] and another reported only the longest sitting bout [56]. The number of prolonged sitting bouts (>30 mins) was reported in two studies [51, 54] while the total duration of prolonged sitting bouts was reported in 3 studies [54, 56, 57]. Four studies reported the number of breaks in sitting [53, 54, 56, 57], while standing and stepping time was reported in four studies [52, 54, 55, 57].

Wear time protocols for activPAL were heterogeneous, with four studies [51–53, 56] requiring continuous wear (24 hours / day), two for waking hours [54, 57] and one for nine hours /day [55], over seven [52, 55–57], ten [53] and fourteen [51, 54] days. Details in S4 Table.

### Secondary outcomes

Six of the seven studies provided some baseline measure of anthropometry, BMI [51–53, 55–57], waist circumference [52, 53], or percentage body fat, [52, 53]. Two studies include baseline measures of blood pressure (BP) [53, 57] or clinical biomarkers [52, 53], including measures of fasting or two-hour blood glucose and/ or insulin, HbA1c, HOMA, cholesterol and triglycerides. Only one study reported follow up of anthropometry or clinical measures, all not significantly different [53]. (S3 Table).

Secondary psychosocial measures were included in only two studies, the EuroQol 5 dimension visual analogue scale (EQ-5D VAS) and hospital anxiety and depression scale (HADS) [53], profile of mood states (POMS) [57].

### Effects of interventions

**Fig 2:**
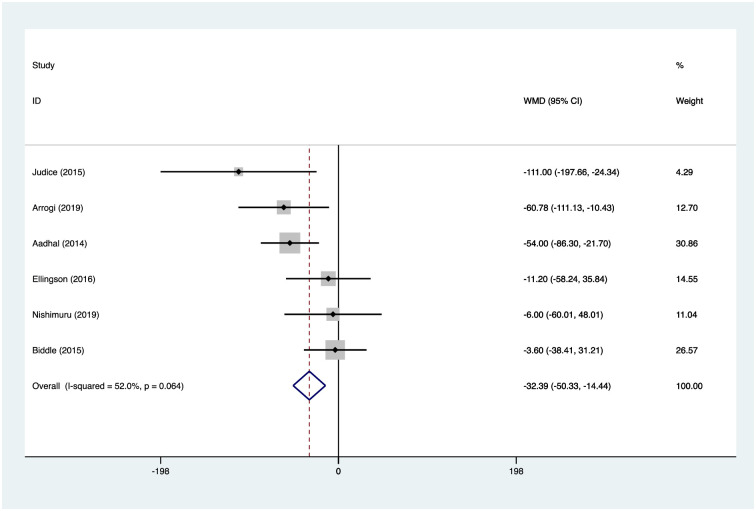
activPAL mean daily sitting time (minutes). H*eterogeneity chi-squared = 10*.*43 (d*.*f*. = *5) p = 0*.*064; I-squared (variation in Weighted Mean Difference (WMD) attributable to heterogeneity) = 52*.*0%; Test of WMD = 0*: *z = 3*.*54 p = 0*.*000*.

**Fig 3:**
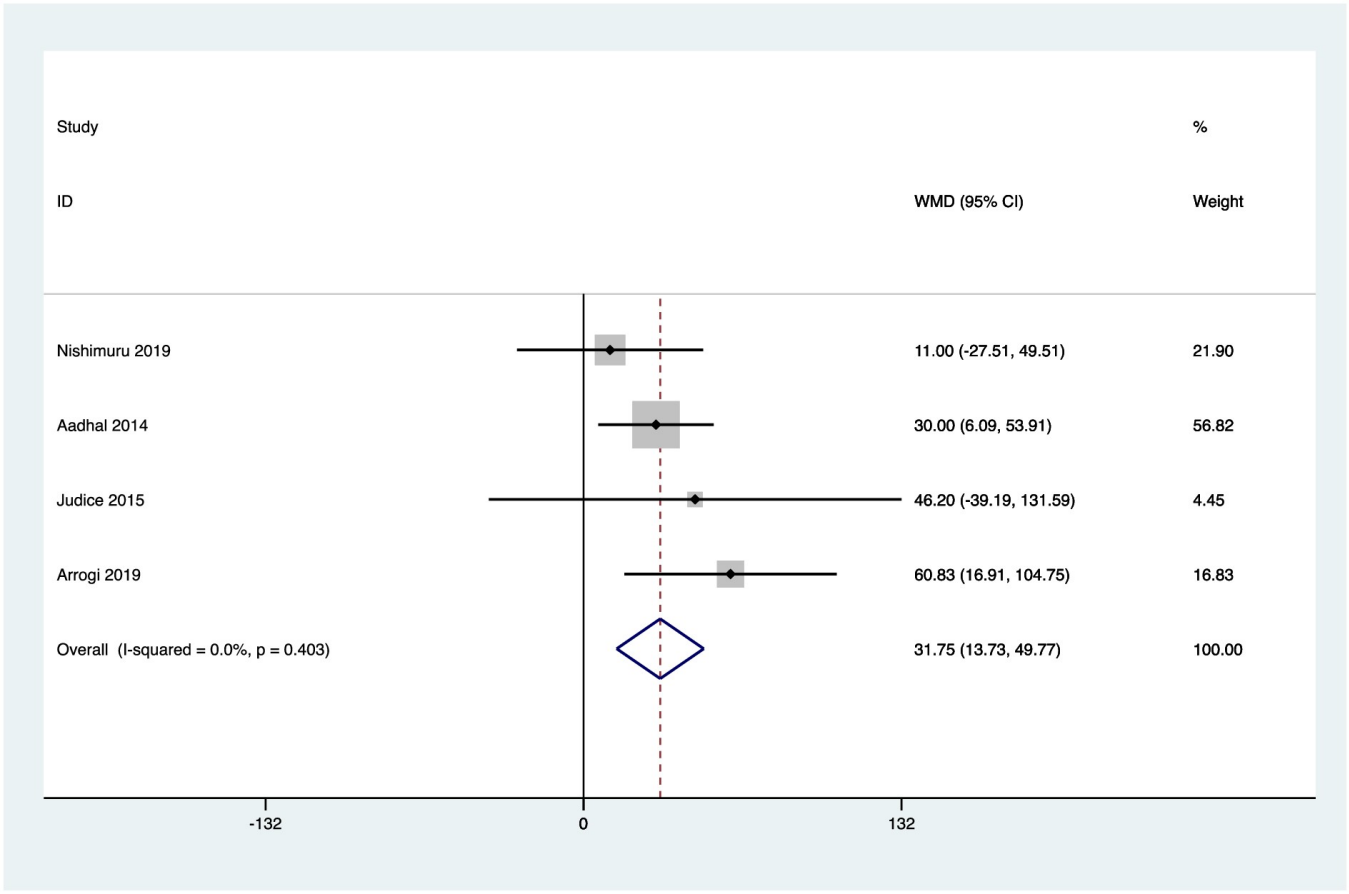
activPAL mean daily standing time (minutes). Heterogeneity chi-squared = 2.93 (d.f. = 3) p = 0.403; I-squared (variation in WMD attributable to heterogeneity) = 0.0%; Test of WMD = 0: z = 3.45 p = 0.001.

**Fig 4:**
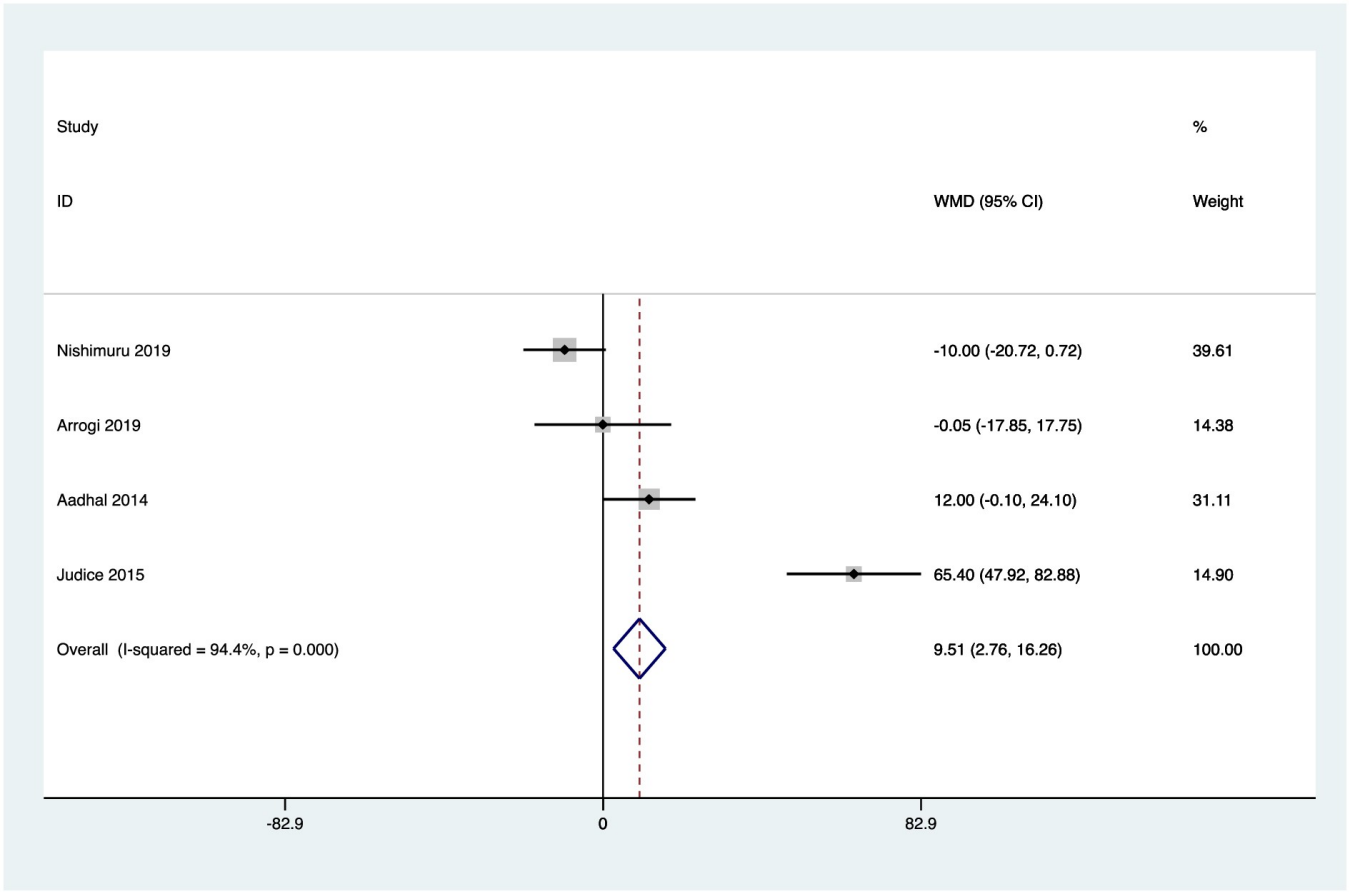
activPAL mean daily stepping time (minutes). Heterogeneity chi-squared = 53.25 (d.f. = 3) p = 0.000; I-squared (variation in WMD attributable to heterogeneity) = 94.4%; Test of WMD = 0: z = 2.76 p = 0.006.

**Fig 5:**
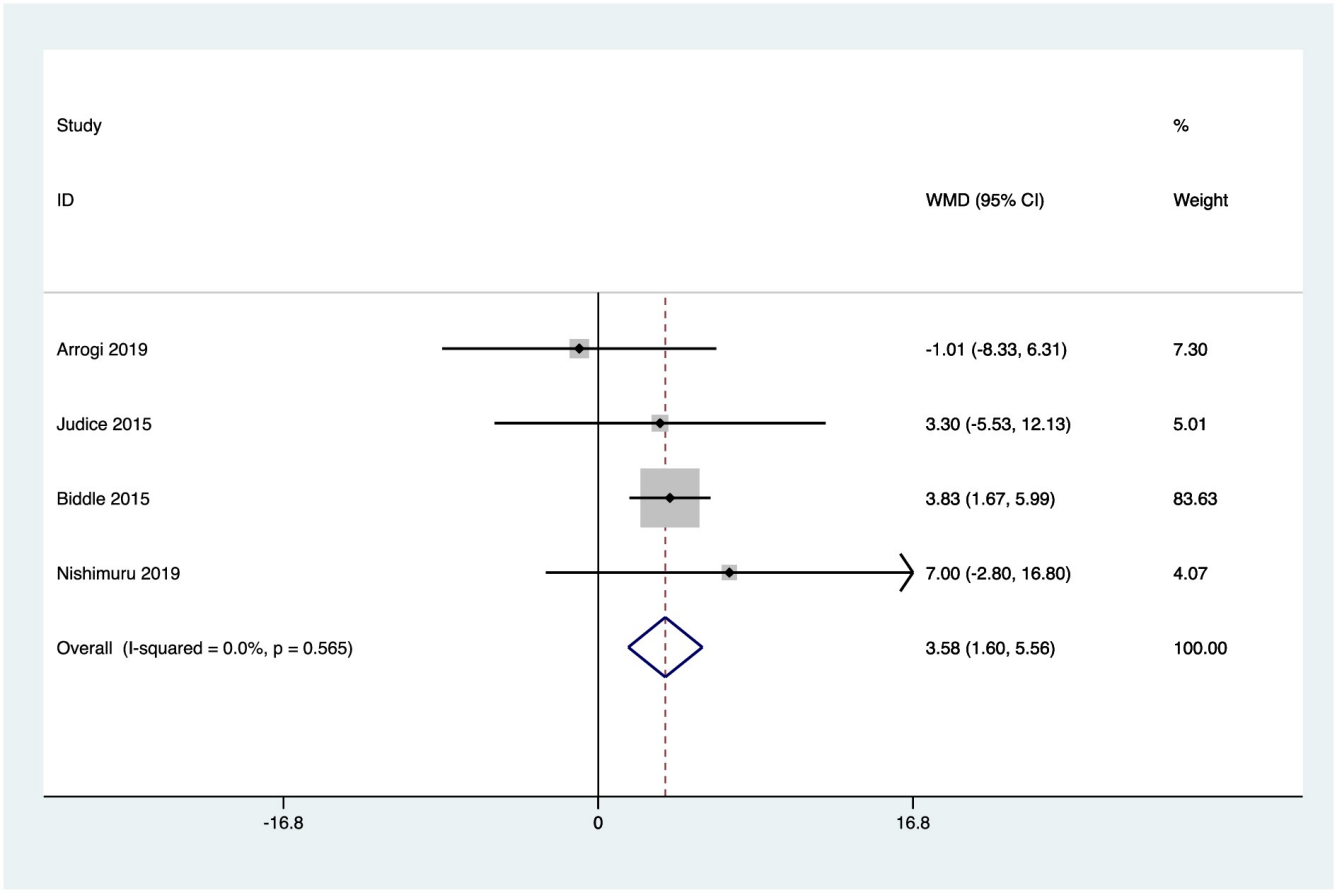
activPAL mean daily rate of sedentary breaks. Heterogeneity chi-squared = 2.04 (d.f. = 3) p = 0.565; I-squared (variation in WMD attributable to heterogeneity) = 0.0%; Test of WMD = 0: z = 3.55 p = 0.000.

Six studies reported activPAL measures of mean daily sitting time [51–55, 57], and four reported mean daily standing time, stepping time [51, 52, 54, 55], and number of sedentary breaks [51, 53–55]. Random effects analysis was performed to calculate weighted mean differences for these outcomes. Pooled analysis revealed a reduction in mean daily sitting time of -32.4mins 95% confidence interval CI (-50.3, -14.4), an increase in mean daily standing time of 31.75mins CI (13.7, 49.8), and mean daily stepping time of 9.5mins CI (2.8, 16.3), and an increased rate of sedentary breaks per day of 3.6 CI (1.6, 5.6). Figs 2–5 illustrate the results.

### Core components of the interventions

#### Interventions: Description and replicability

The TIDieR twelve item checklist [48] was used to score the description and replicability of the intervention and control conditions, with each point scored only if explicitly reported (max score 12). The mean score was 8.1 (SD 1.8) for interventions and 2.7 (SD 1.4) for the control condition (Table 3). All studies reported multiple intervention activities or processes, which varied across the studies. Six of the seven studies reported using a technological component as part of the intervention, as a method to alert prolonged sedentary behaviour, a prompt or cue to interrupt sedentary behaviour and some method of self-monitoring [51, 53–57]. Four of these studies used an accelerometer to deliver a vibrotactile prompt when participants were sedentary for a predetermined time [25–30 mins) [53–55, 57], while one cross-domain study interrupted sedentary behaviour hourly via a computer warning, followed by a locked screen for seven minutes [51]. Two studies used smartphone apps to deliver prompts or cues to interrupt sedentary behaviour and to provide a method of self-monitoring behaviour [54, 56]. Three studies used tailored feedback, delivered either face to face [52], via phone calls and text messages [51] or via a smartphone app [56]. Education about the deleterious effects of sedentary behaviour and the benefits of interrupting sedentary behaviour was reported in only two of the interventions, delivered either face to face in a group [53] or individually via technology embedded at the design phase [54]. One study reported using minimal education as the control condition [57]. Interventions were delivered by ‘research staff’ [54], ‘trained educators’ [53], research nurses [52] and a physical therapist [56] and three did not report this item [51, 55, 57]. Two studies reported individual modification (personalisation) of intervention following review of behavioural goals [52] or monitoring to ensure adherence to daily goals [51]. Four studies reported, strategies to enhance and monitor adherence with intervention [51, 52, 54, 55], three of which also reported, the actual adherence [51, 52, 54] and another study reported only actual adherence [57]. Notably, one further study reported that a key part of the intervention, an education workshop, was ‘very poorly attended’ but did not report on adherence to other parts of the intervention [53].

**Table 3:** Coding for the 12 TIDieR items for individual studies, intervention and control conditions.

Study Year	Group	1.Intervention Name	2.Rationale; Theory; Goal	3.What Materials	4.What Procedures	5.Who Provided	6.How; Mode of Delivery	7.Where	8.When; How much	9.Tailoring	10.Modification during Study	11.How Well; Planned	12.How Well; Actual	Score
Aadahl 2014	Intervention	**✔**	**✔**	**✔**	**✔**	**✔**	**✔**	**✔**	**✔**	**✔**	**✔**	**✔**	**✔**	12
	Control	**✔**	**-**	**-**	**-**	**-**	**-**	**-**	**✔**	**-**	**-**	**-**	**-**	2
Arrogi 2016	Intervention	**✔**	**?**	**✔**	**✔**	**✔**	**✔**	**✔**	**✔**	**?**	**-**	**✔**	**✔**	9
	Control	**✔**	**-**	**-**	**-**	**-**	**-**	**-**	**-**	**-**	**-**	**-**	**-**	1
Biddle 2015	Intervention	**✔**	**✔**	**✔**	**✔**	**✔**	**✔**	**-**	**✔**	**-**	**-**	**-**	**-**	7
	Control	**-**	**-**	**✔**	**-**	**-**	**-**	**-**	**-**	**-**	**-**	**-**	**-**	1
Ellingson 2016	Intervention	**✔**	**✔**	**✔**	**✔**	**-**	**✔**	**?**	**✔**	**✔**	**-**	**-**	**✔**	8
	Control	**✔**	**-**	**-**	**-**	**-**	**-**	**?**	**✔**	**-**	**-**	**-**	**✔**	3
Judice 2015	Intervention	**✔**	**-**	**✔**	**✔**	**?**	**✔**	**?**	**✔**	**✔**	**✔**	**✔**	**✔**	9
	Control	**✔**	**-**	**-**	**✔**	**?**	**?**	**?**	**-**	**-**	**✔**	**✔**	**-**	4
Kitigawa 2020	Intervention SF	**✔**	**-**	**✔**	**✔**	**?**	**✔**	**✔**	**?**	**-**	**-**	**-**	**-**	5
	Intervention TF	**✔**	**?**	**✔**	**✔**	**✔**	**✔**	**✔**	**?**	**✔**	**-**	**-**	**-**	7
	Control	**✔**	**-**	**✔**	**-**	**-**	**-**	**✔**	**-**	**-**	**-**	**-**	**-**	3
Nishimuru 2019	Intervention	**✔**	**-**	**✔**	**✔**	**-**	**✔**	**?**	**✔**	**✔**	**-**	**✔**	**-**	7
	Control	**?**	**-**	**✔**	**✔**	**-**	**✔**	**?**	**✔**	**-**	**-**	**✔**	**-**	5

Notes: description of item **✔** = clear; - = minimal or no description;? = unclear description; SF = self-feedback; TF = tailored feedback. Mean (SD) Intervention 8.0 (1.9); Control 2.7 (1.4)

#### Behaviour change techniques (BCTs) and theory

From the ninety-three BCTs contained in the BCT taxonomy [41], twenty (21.5%) were identified in eight interventions across the seven studies and the number of BCT’s per intervention ranged from three to fourteen (mean 6.6 SD 3.2) representing a total of fifty-three uses of BCTs. The most frequently used BCT was ‘adding objects to the environment’ (predominantly small wearable devices), identified in seven interventions [51, 53–57], while ‘self-monitoring of behaviour’ [51, 53, 55–57], ‘information about health consequences’ [53, 54, 56, 57], and ‘prompts and cues’ were used in five interventions [51, 53–55, 57]. A further three BCTs were used in four interventions, ‘self-monitoring of outcomes of behaviour’ [51, 54, 56], ‘instruction on how to perform the behaviour’ [51, 52, 54, 56] and ‘behaviour substitution‘ [51, 52, 56, 57]. The BCTs identified in the interventions represented eleven of the sixteen BCT Taxonomy hierarchies. Details in Table 4.

**Table 4:** Behaviour change techniques per intervention.

BCT CATEGORY	BCT	AADAHL	ARROGI	BIDDLE	ELLINGSON	JUDICE	KITTIGAWA SF	KITTIGAWA TF	NISHIMURU
**1. GOALS AND PLANNING**	1.1. Goal setting (behaviour)	**✔**				**✔**			
	1.2. Problem solving					**✔**			
	1.4. Action planning					**✔**			
	1.5. Review behaviour goal(s)	**✔**							
**2. FEEDBACK AND MONITORING**	2.2. Feedback on behaviour		**✔**			**✔**			
	2.3. Self-monitoring of behaviour			**✔**	**✔**	**✔**		**✔**	**✔**
	2.4. Self-monitoring of outcomes of behaviour			**✔**		**✔**	**✔**	**✔**	
**3. SOCIAL SUPPORT**	3.1. Social support (unspecified)			**✔**					
	3.2. Social support (practical)	**✔**		**✔**		**✔**			
**4. SHAPING KNOWLEDGE**	4.1. Instruction on how to perform the behaviour	**✔**	**✔**	**✔**		**✔**		**✔**	
**5. NATURAL CONSEQUENCES**	5.1. Information about health consequences		**✔**	**✔**	**✔**		**✔**	**✔**	
**7. ASSOCIATIONS**	7.1. Prompts/cues		**✔**	**✔**	**✔**	**✔**			**✔**
**8. REPETITION AND SUBSTITUTION**	8.2. Behaviour substitution	**✔**			**✔**	**✔**		**✔**	
	8.4. Habit reversal				**✔**	**✔**			
	8.7. Graded tasks		**✔**						
**9. COMPARISON OF OUTCOMES**	9.1. Credible source	**✔**						**✔**	
**10. REWARD AND THREAT**	10.3. Non-specific reward		**✔**						
	10.11. Future punishment					**✔**			
**12. ANTECEDENTS**	12.5. Adding objects to the environment		**✔**	**✔**	**✔**	**✔**	**✔**	**✔**	**✔**
**14. SCHEDULED CONSEQUENCES**	14.2. Punishment					**✔**			

BCTs numbered and categorized according to BCT V1 Taxonomy;

**✔** = BCT present; SF = Self-feedback; TF = Tailored feedback;

A total of eight BCTs were identified across the seven control conditions, although one control condition used no BCTs [52] and five others used only one, either ‘information about health consequences’ [53, 54, 56, 57] or ‘adding objects to the environment’ [55], both are likely to be active ingredients and to attenuate control group sedentary behaviour. The mean number of BCTs identified per control group was 1.7 (SD 2.2). One study [51] used considerably more BCTs than any other study, both for intervention (n = 14) and control (n = 7) conditions. Details in Table 5.

**Table 5:** Behaviour change techniques per control group.

BCT CATEGORY	BCT	AADAHL	ARROGI	BIDDLE	ELLINGSON	JUDICE	KITTIGAWA	NISHIMURU
**1. GOALS AND PLANNING**	1.1. Goal setting (behaviour)					**✔**		
**2. FEEDBACK AND MONITORING**	2.3. Self-monitoring of behaviour					**✔**		
	2.4. Self-monitoring of outcomes of behaviour					**✔**		
**3. SOCIAL SUPPORT**	3.2. Social support (practical)					**✔**		
**4. SHAPING KNOWLEDGE**	4.1. Instruction on how to perform the behaviour					**✔**		
**5. NATURAL CONSEQUENCES**	5.1. Information about health consequences		**✔**	**✔**	**✔**		**✔**	
**7. ASSOCIATIONS**	7.1. Prompts/cues					**✔**		
**12. ANTECEDENTS**	12.5. Adding objects to the environment					**✔**		**✔**

BCTs numbered and categorized according to BCT V1 Taxonomy; **✔** = BCT present;

There was limited use of theory to inform the interventions, specifically only three studies reported any behaviour change theory as identified by the TIDieR analysis [52, 53, 57]. Those theories were habit theory of behaviour change [57], behaviour choice theory [53], common sense model dual process theory [53] and social cognitive theory [53]. However, evidence for application of the theories was lacking, with no explicit or hypothesised links to BCTs or intervention processes reported.

#### Effectiveness of intervention components, behavioural theory and BCTs

Due to heterogeneity in content and small number of studies, statistical analysis of effectiveness relative to intervention components, BCTs or theory was not possible. Study length was very short (2 weeks) in two of the most effective interventions [51, 54] but considerably longer in the third most effective intervention (26 weeks) [52]. Whilst the longest study [53], reported the least comparative difference between intervention and control groups at follow up (52 weeks), than any of the other studies included in the review. This study which was also the largest study and therefore had the greatest weight in the effects analysis, reported that the mean daily sitting time was reduced in both the intervention (-38.4 mins) and control (-34.8 mins) groups. Despite its robust methodology and design, the study had an attrition rate of almost thirty-three percent, and an integral part of the intervention (three-hour education workshop) was very poorly attended. Furthermore, the control group received a pamphlet which contained some of the key educational components delivered in the intervention group workshop, and this may account for attenuating sedentary behaviour within the control group. The true intervention effect size in this study may have been reduced, by attrition, compliance and control contamination.

Conversely, the smallest study (n = 10) [51] may be underpowered in its effect size, but if results can be replicated in a larger population, -111 minutes reduction in mean daily sitting time, this intervention has the potential to exceed the reductions in sedentary behaviour reported by Prince et al [32].

### Study quality

Only one study was assessed as having a low risk of selection bias with evidence of both random sequence generation and allocation concealment [55]. Allocation was concealed by issuing the activPAL device, to both control and intervention groups for ‘postural assessment’, essentially acting as placebo for the control group, whilst only the intervention group received vibrotactile feedback from the device. Four further studies had a moderate risk of selection bias with evidence of random sequence generation but not allocation concealment [51, 52, 56, 57] and two studies had evidence of neither and were deemed high risk of selection bias [53, 54].

Risk of performance bias (participant and personnel blinding) was determined to be high for all of the included studies except one [56] which reported blinding of personnel and therefore risk was assessed as moderate. Only one study reported blinding of participants but this was assessed moderate risk due to the nature of the intervention being apparent to participants [55]. All six other studies reported that it was not possible to blind the participants. Two studies report that study personnel were aware of the allocation [52, 57] and four did not report personnel blinding [51, 53–55].

Four studies were considered low risk for detection bias with blinding of outcome assessment reported, [52, 53, 56, 57] while three studies did not report this blinding and were assessed as high risk of detection bias [51, 54, 55].

**Fig 6:**
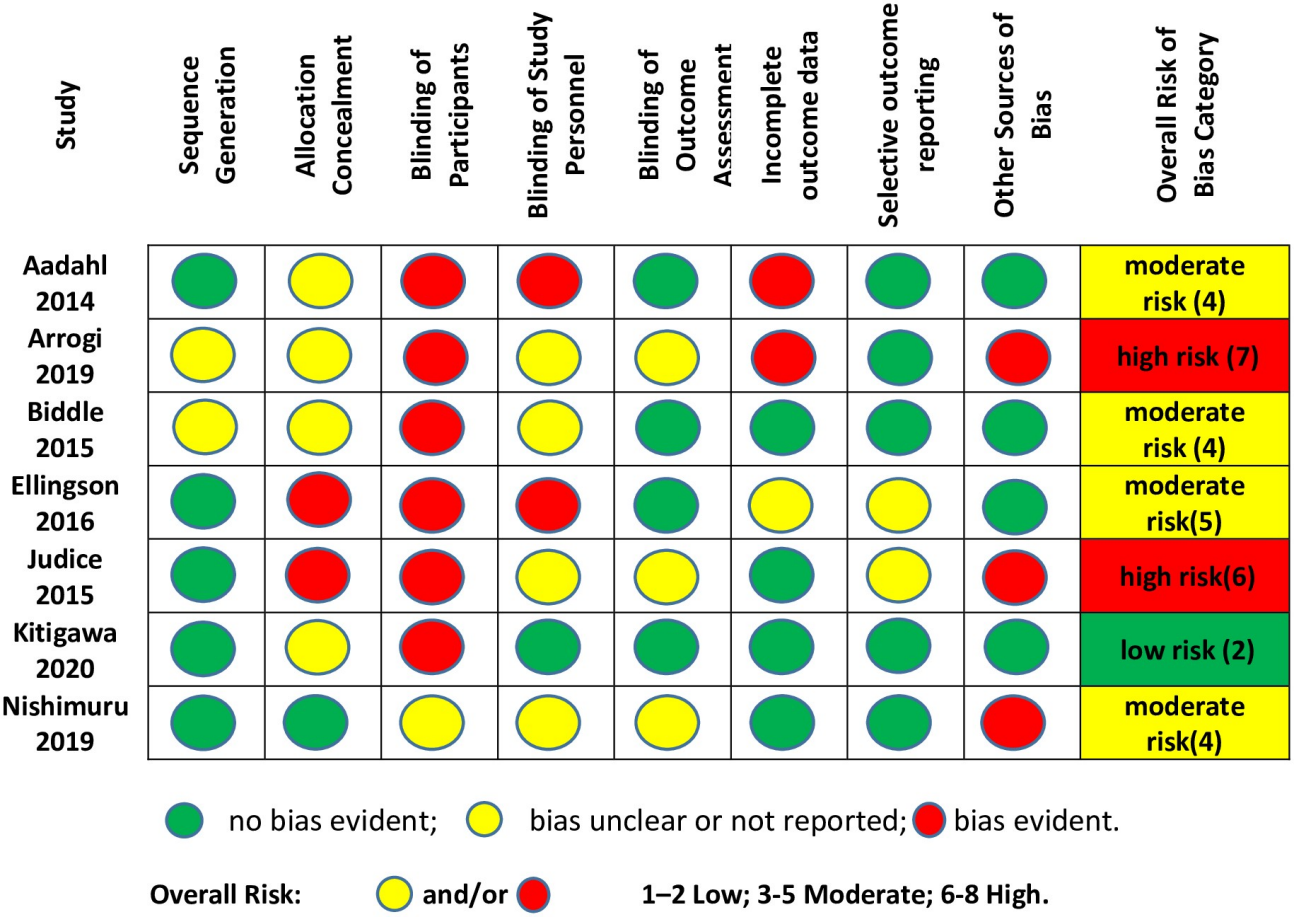
Risk of bias summary.

Four studies who reported intention to treat analysis, were considered low risk for attrition bias [51, 53, 55, 56], while three studies were considered high risk for attrition bias [52, 54, 57] due to incomplete outcome data, reported as ‘missing at random’ and where complete case analysis only was performed. Two studies were assessed as moderate risk for reporting bias due to missing between group analysis [57] or selective reporting of means, and missing pre and post outcome measures [51]. The five other studies were assessed as low risk for reporting bias with all primary outcome measures reported [52–56]. Three studies were identified as high risk for other sources of bias, due to no reported sample size calculation [54], sample size based on a clinical trial measuring a different outcome [55] and no crossover washout and very small sample size [51]. Four studies were considered low risk for other bias, reporting sample size calculations, ethical approval, funding sources and sensitivity analysis [52, 53, 56, 57]. Overall risk of bias was determined to be low for only one study [56], while four studies were assessed as moderate risk of overall bias [52, 53, 55, 57] and two studies were assessed as high risk of bias [51, 54]. A summary of the risk of bias across the included studies is shown in Fig 6.

## Discussion

### Intervention effectiveness

The results of this systematic review suggest that interventions designed primarily and solely to reduce sedentary behaviour, can effectively reduce total daily sitting time in the short term by, on average 32 minutes per day, although heterogeneity in design, content, and population exists. This is an encouraging finding, because this reduction in sedentary time is likely to have clinical benefits in sedentary people [7, 58–60]. The odds ratio per additional hour of sedentary time is reported to be between 1.09 and 1.22 for development of metabolic syndrome, and 1.39 for type 2 diabetes, representing a linear relationship, and suggesting that overall reduction of 32 minutes sedentary behaviour per day will have a positive clinical effect [58–60]. Further experimental research, will be beneficial to accurately understand the clinical implications of sedentary behaviour reduction, and to determine long term sustainability of the behavioural change.

In a subset of studies included in the meta-analysis, there is an equivalent increase in standing time of 32 minutes / day, suggesting that reductions in sitting are largely achieved by standing. Replacement of sitting with standing, which has been reported in studies of workplace interventions [29, 61, 62]. This represents another positive outcome, since replacing prolonged sitting (>30mins) with standing improves insulin sensitivity, glucose control [63] and cardio-metabolic markers [64, 65]. These improvements, if maintained in the long term, will reduce morbidity and mortality.

The reduction in mean daily sitting time reported in this meta-analysis is considerably less than reported by Prince et al [32] for interventions designed specifically to interrupt sedentary behaviour (91 minutes), and is closer to the reduction reported for non-specific sedentary behaviour interventions (35 minutes) in a number of other reviews [32, 35]. Potential reasons for this difference in findings include factors intrinsic to the studies included in this review, which have been identified in the Results, namely; contamination of the control condition, poor attendance or adherence to intervention, attrition, under estimation of effect due to small sample size, and high risk of bias for two of the most effective studies. [51, 54]. Other potential reasons for the difference in findings arise from the different inclusion criteria for both reviews, resulting in no overlap of studies. The eight studies in the Prince et al review were excluded from this review for the following reasons; three studies were non-randomised [66–68], six studies were workplace studies [21, 46, 66–69] and two included other primary outcomes, i.e. cardio-metabolic risk factors [70] and energy intake and expenditure [71]. The predominance of workplace interventions is likely to contribute to the difference. Whilst this review includes only one study which has a workplace component [51], the reduction in sitting time exceeded that of the Prince et al review (-111mins vs -91mins). It is likely that the workplace component was integral to the magnitude of the effect, and whilst leisure time was identified by self report, as the domain to best achieve sitting time reductions, this is not substantiated with device measures. It is necessary to design interventions with both workplace and leisure time components, to reduce overall sedentary behaviour and its consequences.

### Core intervention components, tidier, behaviour theories and BCTs

While the studies and interventions were heterogeneous, a number of similarities were identified both in interventions and BCTs employed, with most interventions using wearable or personal technology, which combined a number of BCTs (e.g. adding objects to the environment, self-monitoring, prompts and cues). Wearable technology as a measurement tool, particularly if feedback is provided, may in itself, influence sedentary behaviour, since wearing a pedometer has been found to increase daily steps [72], making estimates of intervention effect challenging. Nevertheless, real time vibrotactile feedback via wearable or personal technology has been identified as potentially effective for delivering BCTs across all domains, and is used by four studies in this review [53–55, 57]. Its potency as an active ingredient is questionable since it is identified in only one [54] of the three most effective studies [51, 52, 54]. Suggested active ingredients, used exclusively in two of the three most effective studies, are ‘goal setting’ and ‘feedback on behaviour’. The feedback provided in these studies was delivered by a person, either face to face or via phone-calls and personal texts, suggesting that human input is required in addition to technology, or that technology need to be further enhanced and personalised. Further research to identify the essential human /social components of interventions, and the development of enhanced personalised technology may bridge this gap.

The theory of additive effects of linking BCT’s [44] is supported by the finding that the most effective intervention [51] contains considerably more BCTs (n = 14), than the average number across the studies (n = 6.6), and four are used exclusively in that study (problem solving, action planning, future punishment and punishment). Moreover, some of the active ingredients of interventions, particularly the technology components may be under-reported [73]. For example, ‘feedback on behaviour’ is likely to be present in interventions named ‘self-feedback’ and ‘tailored feedback’ [56] but in line with BCT V1 taxonomy, it is not coded unless it is explicitly reported, identifying the need for better reporting of BCT’s.

Whilst a number of interventions were theory inspired, none systematically linked theory to the application of the intervention, which is necessary to draw accurate effect correlations [74, 75]. Ongoing research to develop a consensus framework for identifying hypothesised links between intervention content, mechanisms of action and behavioural theories [44], must also evaluate the application of interventions to draw accurate conclusions and to enhance understanding of the active components of interventions and their effective theories.

Furthermore, adherence to the TIDieR guidelines may enhance effectiveness, since the three most effective interventions [51, 52, 54] also scored most highly in the TIDieR coding. However, In line with Hoffman’s [48] assertion, the control conditions are particularly poorly reported in all seven studies. The use of the TIDieR checklist at the design phase of a trial, for both the intervention and the control conditions has the potential to improve not only the replicability of the intervention, but also to ensure identification of the effective components of the intervention, and to pre-empt and thereby limit the potential contamination or attenuation of the control condition, and ensure a more reliable estimate of effect size.

### Strengths and limitations

This systematic review synthesises evidence from interventions designed specifically to target change in overall sedentary behaviour. A rigorous methodology from search strategy to data coding, extraction, analysis and reporting was used. Other reviews to date have included interventions designed to change PA, with sedentary behaviour a secondary outcome [35, 38, 39]. The criteria for inclusion in this review were deliberately narrow in order to find the most efficacious interventions for reducing sedentary behaviour. However, this exposes the paucity of studies actually targeting change in sedentary behaviour, despite the apparent wealth of research reporting on sedentary behaviour. A number of studies were excluded when rigourous screening of supplementary data or prior publications, revealed that interventions were not primarily targeting sedentary behaviour change. Researchers must explicitly report when sedentary behaviour change is the primary outcome and target of an intervention, and when it is not.

Thus the small number of studies available, limits the generalisability and power of the findings. Furthermore, the sample sizes of the studies were generally small with four pilot studies included and therefore, potential for underestimation of the actual effect, and the relatively short follow up period of the outcome measurements of the studies prevents the analysis of the important longevity of the effect. Also, of note, measurement and reporting of change in sedentary behaviour and secondary outcomes was not standardised across the studies and limited the meta-analysis. The development of a core set of outcome measures for sedentary behaviour research will enhance future meta-analyses.

Finally, the quality of the evidence was low to moderate, perhaps in part due to lack of reporting, although for all studies, it was not possible to blind the participants due to the intervention design. However, future studies need to be of higher quality with rigorous reporting of sources of bias.

## Conclusion

In summary, although limited by small sample sizes and short follow up periods, this review suggests that interventions primarily and solely designed to reduce sedentary behaviour can reduce overall daily sitting time and increase standing time by half an hour, in the short to medium term. Effective characteristics and behaviour change strategies are identified for use in the development of future high quality interventions targeting sedentary behaviour change. The most potent BCTs, or active ingredients, identified by the review are ‘goal setting behaviour’ and ‘feedback on behaviour’, whilst intervention fidelity and delivery of content will be improved by the TIDieR components ‘planning and implementing strategies to measure and enhance adherence to the intervention’.

## Supporting information

S1 Table Search terms. (DOCX)

S2 Table Studies excluded and reasons. Full-text articles excluded (n = 38) reasons; workplace (n = 18); SB not sole primary target of intervention (n = 8); older adult (n = 1); workplace *and* SB not sole primary target of intervention (n = 5); workplace *and* not randomised (n = 1); workplace *and* SB not sole primary target of intervention *and* not randomised (n = 2); SB not sole primary target of intervention *and* older adults (n = 3);.
(DOCX)

S3 Table Anthropometric measures and biomarkers. Note; Only Biddle reported any follow up measures; SF = self feedback; TF = tailored feedback.
(DOCX)

S4 Table activPAL wear time protocol and criteria for inclusion in analysis. (DOCX)

S1 Checklist (DOC)
