# Supplementary material for: Efficacy, characteristics, behavioural models and behaviour change strategies, of non-workplace interventions specifically targeting sedentary behaviour; a systematic review and meta-analysis of randomised control trials in healthy ambulatory adults
Source: PLoS One. 2021 Sep 7;16(9):e0256828. doi: 10.1371/journal.pone.0256828 (PMC8423252; doi:10.1371/journal.pone.0256828)
Supplement: S1 Table — (DOCX) [file pone.0256828.s001.docx]

# **S1 Table Search terms**

| **1 . Pubmed Search** SearchActionsDetailsQueryResultsTime#11  Search: **#7** Filters: **Humans, English, from 2000 – 2020** [5,700](https://pubmed.ncbi.nlm.nih.gov/?term=%237&filter=species.humans&filter=language.english&filter=years.2000-2020&sort=relevance)  Search: **#7** Filters: **English, Humans** [6,871](https://pubmed.ncbi.nlm.nih.gov/?term=%237&filter=language.english&filter=species.humans&sort=relevance)  Search: **#7** Filters: **English** [7,477](https://pubmed.ncbi.nlm.nih.gov/?term=%237&filter=language.english&sort=relevance)  Search: **#7** [7,611](https://pubmed.ncbi.nlm.nih.gov/?term=%237&sort=)  Search: **((Sedentary OR Sedentary Behaviour OR Sedentary Behavior OR Sedentary lifestyle Or Sedentary time OR Sedentariness OR Physical inactivity Or Physically inactive OR Physically Underactive Or Under Active OR low energy expenditure OR <1.5 MET OR (Prolonged AND (Sitting OR Reclined Or Reclining OR Seated OR Stationary) OR Sedentary Time OR Sedentary Bouts OR Sedentary Breaks) AND (Randomised Controlled trial OR Randomized controlled Trial OR Randomised Clinical trial OR randomized clinical trial OR Randomised trial OR Randomized trial OR Controlled clinical trial OR Controlled Trial OR control trial OR RCT OR RCTS or RCT’s)) AND (Adult OR Adults OR Men OR Women OR Individuals OR Employees OR Patients OR Seniors OR Older adult* OR Elderly NOT (Child OR Children Or Adolescent Or Teenager Or Teen))**  [7,611](https://pubmed.ncbi.nlm.nih.gov/?term=%28%28Sedentary+OR+Sedentary+Behaviour+OR+Sedentary+Behavior+OR+Sedentary+lifestyle+Or+Sedentary+time+OR+Sedentariness+OR+Physical+inactivity+Or+Physically+inactive+OR+Physically+Underactive+Or+Under+Active+OR+low+energy+expenditure+OR+%3C1.5+MET+OR+%28Prolonged+AND+%28Sitting+OR+Reclined+Or+Reclining+OR+Seated+OR+Stationary%29+OR+Sedentary+Time+OR+Sedentary+Bouts+OR+Sedentary+Breaks%29+AND+%28Randomised+Controlled+trial+OR+Randomized+controlled+Trial+OR+Randomised+Clinical+trial+OR+randomized+clinical+trial+OR+Randomised+trial+OR+Randomized+trial+OR+Controlled+clinical+trial+OR+Controlled+Trial+OR+control+trial+OR+RCT+OR+RCTS+or+RCT%E2%80%99s%29%29+AND+%28Adult+OR+Adults+OR+Men+OR+Women+OR+Individuals+OR+Employees+OR+Patients+OR+Seniors+OR+Older+adult%2A+OR+Elderly+NOT+%28Child+OR+Children+Or+Adolescent+Or+Teenager+Or+Teen%29%29&sort=)  Search: **Adult OR Adults OR Men OR Women OR Individuals OR Employees OR Patients OR Seniors OR Older adult* OR Elderly NOT (Child OR Children Or Adolescent Or Teenager Or Teen)** [11,290,680](https://pubmed.ncbi.nlm.nih.gov/?term=Adult+OR+Adults+OR+Men+OR+Women+OR+Individuals+OR+Employees+OR+Patients+OR+Seniors+OR+Older+adult%2A+OR+Elderly+NOT+%28Child+OR+Children+Or+Adolescent+Or+Teenager+Or+Teen%29&sort=relevance)  Search: **Randomised Controlled trial OR Randomized controlled Trial OR Randomised Clinical trial OR randomized clinical trial OR Randomised trial OR Randomized trial OR Controlled clinical trial OR Controlled Trial OR control trial OR RCT OR RCTS or RCT’s** [1,112,489](https://pubmed.ncbi.nlm.nih.gov/?term=Randomised+Controlled+trial+OR+Randomized+controlled+Trial+OR+Randomised+Clinical+trial+OR+randomized+clinical+trial+OR+Randomised+trial+OR+Randomized+trial+OR+Controlled+clinical+trial+OR+Controlled+Trial+OR+control+trial+OR+RCT+OR+RCTS+or+RCT%E2%80%99s&sort=relevance)  Search: **Sedentary OR Sedentary Behaviour OR Sedentary Behavior OR Sedentary lifestyle Or Sedentary time OR Sedentariness OR Physical inactivity Or Physically inactive OR Physically Underactive Or Under Active OR low energy expenditure OR <1.5 MET OR (Prolonged AND (Sitting OR Reclined Or Reclining OR Seated OR Stationary) OR Sedentary Time OR Sedentary Bouts OR Sedentary Breaks** [15,254](https://pubmed.ncbi.nlm.nih.gov/?term=Sedentary+OR+Sedentary+Behaviour+OR+Sedentary+Behavior+OR+Sedentary+lifestyle+Or+Sedentary+time+OR+Sedentariness+OR+Physical+inactivity+Or+Physically+inactive+OR+Physically+Underactive+Or+Under+Active+OR+low+energy+expenditure+OR+%3C1.5+MET+OR+%28Prolonged+AND+%28Sitting+OR+Reclined+Or+Reclining+OR+Seated+OR+Stationary%29+OR+Sedentary+Time+OR+Sedentary+Bouts+OR+Sedentary+Breaks&sort=relevance) |
| --- |

| **2. EMBASE Search** **#7**  **#6**  #1 AND #2 AND #3 AND #4 AND [english]/lim AND [2000-2020]/py [808](https://www-embase-com.ucd.idm.oclc.org/) **#5**  #1 AND #2 AND #3 AND #4 [809](https://www-embase-com.ucd.idm.oclc.org/) **#4**  (**'randomised controlled trial'**:ti,ab,kw OR **'randomized controlled trial'**:ti,ab,kw OR **'randomised clinical trial'**:ti,ab,kw OR **'randomized clinical trial'**:ti,ab,kw OR **'randomised trial'**:ti,ab,kw OR **'randomized trial'**:ti,ab,kw OR **'controlled clinical trial'**:ti,ab,kw OR **'controlled trial'**:ti,ab,kw OR **'control trial'**:ti,ab,kw OR **rct**:ti,ab,kw OR **rcts**:ti,ab,kw) AND [2000-2020]/py [315,801](https://www-embase-com.ucd.idm.oclc.org/) **#3**  (**adult**:ti,ab,kw OR **adults**:ti,ab,kw OR **men**:ti,ab,kw OR **women**:ti,ab,kw OR **individuals**:ti,ab,kw OR **employees**:ti,ab,kw OR **patients**:ti,ab,kw OR **seniors**:ti,ab,kw OR **'older adult*'**:ti,ab,kw OR **elderly**:ti,ab,kw) NOT (**child**:ti,ab,kw OR **children**:ti,ab,kw OR **adolescent**:ti,ab,kw OR **teenager**:ti,ab,kw OR **teen**:ti,ab,kw) [9,989,090](https://www-embase-com.ucd.idm.oclc.org/) **#2**  (**'sedentary behavior'**:ti,ab,kw OR **'sedentary behaviour'**:ti,ab,kw OR **'sedentary lifestyle'**:ti,ab,kw OR **sedentariness**:ti,ab,kw OR **'physical inactivity'**:ti,ab,kw OR **'physically inactive'**:ti,ab,kw OR **'physically underactive'**:ti,ab,kw OR **'underactive lifestyle'**:ti,ab,kw OR **'low energy expenditure'**:ti,ab,kw OR **1.5met**:ti,ab,kw OR **'prolonged sitting'**:ti,ab,kw OR **'prolonged reclining'**:ti,ab,kw OR **'sitting time'**:ti,ab,kw OR **'time seated'**:ti,ab,kw OR **'sedentary time'**:ti,ab,kw OR **'sedentary breaks'**:ti,ab,kw OR **'sedentary bouts'**:ti,ab,kw) AND [2000-2020]/py [24,673](https://www-embase-com.ucd.idm.oclc.org/) **#1**  (**'sedentary behavior'**:ti,ab,kw OR **'sedentary behaviour'**:ti,ab,kw OR **'sedentary lifestyle'**:ti,ab,kw OR **sedentariness**:ti,ab,kw OR **'physical inactivity'**:ti,ab,kw OR **'physically inactive'**:ti,ab,kw OR **'physically underactive'**:ti,ab,kw OR **'underactive lifestyle'**:ti,ab,kw OR **'low energy expenditure'**:ti,ab,kw OR **1.5met**:ti,ab,kw OR **'prolonged sitting'**:ti,ab,kw OR **'prolonged reclining'**:ti,ab,kw OR **'sitting time'**:ti,ab,kw OR **'time seated'**:ti,ab,kw OR **'sedentary time'**:ti,ab,kw OR **'sedentary breaks'**:ti,ab,kw OR **'sedentary bouts'**:ti,ab,kw) AND [2000-2020]/py |
| --- |
| **3 Cochrane Central Register Search**109 **Cochrane Reviews matching**Sedentary Behaviour OR Sedentary Behavior OR Sedentary lifestyle Or Sedentary time OR Sedentariness OR Physical inactivity Or Physically inactive OR Physically Underactive Or Underactive lifestyle OR low energy expenditure OR <1.5 MET OR Prolonged Sitting OR prolonged reclining OR Sitting time OR Sedentary Bouts OR Sedentary Breaks in Title Abstract Keyword AND Adult OR Adults OR Men OR Women OR Individuals OR Employees OR Patients OR Seniors OR Elderly in Title Abstract Keyword NOT Child OR Children OR Infant OR Adolescent OR Teenager OR Teen in Title Abstract Keyword AND Randomised Controlled trial OR Randomized controlled Trial OR Randomised Control trial OR Randomized control Trial OR Randomised Clinical trial OR randomized clinical trial OR Randomised trial OR Randomized trial OR Controlled clinical trial OR Controlled Trial OR control trial OR RCT OR RCTS or RCT’s in Title Abstract Keyword - (Word variations have been searched) [**Cochrane Database of Systematic Reviews**](https://www.cochranelibrary.com/) 6853 **Trials matching**Sedentary Behaviour OR Sedentary Behavior OR Sedentary lifestyle Or Sedentary time OR Sedentariness OR Physical inactivity Or Physically inactive OR Physically Underactive Or Underactive lifestyle OR low energy expenditure OR <1.5 MET OR Prolonged Sitting OR prolonged reclining OR Sitting time OR Sedentary Bouts OR Sedentary Breaks in Title Abstract Keyword AND Adult OR Adults OR Men OR Women OR Individuals OR Employees OR Patients OR Seniors OR Elderly in Title Abstract Keyword NOT Child OR Children OR Infant OR Adolescent OR Teenager OR Teen in Title Abstract Keyword AND Randomised Controlled trial OR Randomized controlled Trial OR Randomised Control trial OR Randomized control Trial OR Randomised Clinical trial OR randomized clinical trial OR Randomised trial OR Randomized trial OR Controlled clinical trial OR Controlled Trial OR control trial OR RCT OR RCTS or RCT’s in Title Abstract Keyword - (Word variations have been searched) |
| **4 CINAHL Search**  \|  \| **Search Terms** \| **Search Options** \| **Actions** \| \| --- \| --- \| --- \| --- \| \|  \| S4 \| S1 AND S2 AND S3 \| **Limiters** - Publication Year: 2000-2020; English Language; Human  **Expanders** - Apply equivalent subjects  **Search modes** - Boolean/Phrase \| [**View Results**](javascript:__doPostBack('ctl00$ctl00$FindField$FindField$historyControl$HistoryRepeater$ctl00$linkResults','')) (10,129) \| \|  \| S3 \| Adult OR Adults OR Men OR Women OR Individuals OR Employees OR Patients OR Seniors OR Older adult* OR Elderly NOT (Child OR Children Or Adolescent Or Teenager Or Teen) \| **Expanders** - Apply equivalent subjects  **Search modes** - Boolean/Phrase \| [**View Results**](javascript:__doPostBack('ctl00$ctl00$FindField$FindField$historyControl$HistoryRepeater$ctl01$linkResults','')) (2,938,822) \| \|  \| S2 \| Randomised Controlled trial OR Randomized controlled Trial OR Randomised Clinical trial OR randomized clinical trial OR Randomised trial OR Randomized trial OR Controlled clinical trial OR Controlled Trial OR control trial OR RCT OR RCTS or RCT’s \| **Expanders** - Apply equivalent subjects  **Search modes** - Boolean/Phrase \| [**View Results**](javascript:__doPostBack('ctl00$ctl00$FindField$FindField$historyControl$HistoryRepeater$ctl02$linkResults','')) (215,837) \| \|  \| S1 \| Sedentary OR Sedentary Behaviour OR Sedentary Behavior OR Sedentary lifestyle Or Sedentary time OR Sedentariness OR Physical inactivity Or Physically inactive OR Physical* Inactivit* OR Underactive Or Under Active OR low energy expenditure OR <1.5 MET OR Prolonged Sitting OR Sit* OR Reclin* OR Seated OR Stationary OR Sitting OR Sedentary Time OR Sedentary Bouts OR Sedentary Breaks \| **Expanders** - Apply equivalent subjects  **Search modes** - Boolean/Phrase \| [**View Results**](javascript:__doPostBack('ctl00$ctl00$FindField$FindField$historyControl$HistoryRepeater$ctl03$linkResults','')) (239,344 \| |
| **5 PsychINFO search**  \| Select item 4 \| **S4** \| [(Sedentary OR Sedentary Behaviour OR Sedentary Behavior OR Sedentary lifestyle Or Sedentary time OR Sedentariness OR Physical inactivity Or Physically inactive OR Physical* Inactivit* OR Underactive Or Under Active OR low energy expenditure OR <1.5 MET OR Prolonged Sitting OR Sit* OR Reclin* OR Seated OR Stationary OR Sitting OR Sedentary Time OR Sedentary Bouts OR Sedentary Breaks) AND (Adult OR Adults OR Men OR Women OR Individuals OR Employees OR Patients OR Seniors OR Older adult* OR Elderly NOT (Child OR Children Or Adolescent Or Teenager Or Teen)) AND (Randomised Controlled trial OR Randomized controlled Trial OR Randomised Clinical trial OR randomized clinical trial OR Randomised trial OR Randomized trial OR Controlled clinical trial OR Controlled Trial OR control trial OR RCT OR RCTS or RCT’s Randomised Controlled trial OR Randomized controlled Trial OR Randomised Clinical trial OR randomized clinical trial OR Randomised trial OR Randomized trial OR Controlled clinical trial OR Controlled Trial OR control trial OR RCT OR RCTS or RCT’s andomised Controlled trial OR Randomized controlled Trial OR Randomised Clinical trial OR randomized clinical trial OR Randomised trial OR Randomized trial OR Controlled clinical trial OR Controlled Trial OR control trial OR RCT OR RCTS or RCT’s)](https://search.proquest.com/recentsearches.recentsearchtabview.recentsearchesgridview.scrolledrecentsearchlist.checkdbssearchlink:rerunsearch/1C5FA48FE5EF41B5PQ/None?site=psycinfo&t:ac=RecentSearches) \| [**4,129**](https://search.proquest.com/recentsearches.recentsearchtabview.recentsearchesgridview.scrolledrecentsearchlist.checkdbssearchlink_0:rerunsearch/1C5FA48FE5EF41B5PQ/None?site=psycinfo&t:ac=RecentSearches) \| \| --- \| --- \| --- \| --- \| \| Select item 3 \| **S3** \| [Randomised Controlled trial OR Randomized controlled Trial OR Randomised Clinical trial OR randomized clinical trial OR Randomised trial OR Randomized trial OR Controlled clinical trial OR Controlled Trial OR control trial OR RCT OR RCTS or RCT’s Randomised Controlled trial OR Randomized controlled Trial OR Randomised Clinical trial OR randomized clinical trial OR Randomised trial OR Randomized trial OR Controlled clinical trial OR Controlled Trial OR control trial OR RCT OR RCTS or RCT’s andomised Controlled trial OR Randomized controlled Trial OR Randomised Clinical trial OR randomized clinical trial OR Randomised trial OR Randomized trial OR Controlled clinical trial OR Controlled Trial OR control trial OR RCT OR RCTS or RCT’s](https://search.proquest.com/recentsearches.recentsearchtabview.recentsearchesgridview.scrolledrecentsearchlist.checkdbssearchlink:rerunsearch/52FB026606FD4CB4PQ/None?site=psycinfo&t:ac=RecentSearches) \| [**102,464**](https://search.proquest.com/recentsearches.recentsearchtabview.recentsearchesgridview.scrolledrecentsearchlist.checkdbssearchlink_0:rerunsearch/52FB026606FD4CB4PQ/None?site=psycinfo&t:ac=RecentSearches) \| \| Select item 2 \| **S2** \| [Adult OR Adults OR Men OR Women OR Individuals OR Employees OR Patients OR Seniors OR Older adult* OR Elderly NOT (Child OR Children Or Adolescent Or Teenager Or Teen)](https://search.proquest.com/recentsearches.recentsearchtabview.recentsearchesgridview.scrolledrecentsearchlist.checkdbssearchlink:rerunsearch/B905A9A598C64F50PQ/None?site=psycinfo&t:ac=RecentSearches) \| [**2,191,839**](https://search.proquest.com/recentsearches.recentsearchtabview.recentsearchesgridview.scrolledrecentsearchlist.checkdbssearchlink_0:rerunsearch/B905A9A598C64F50PQ/None?site=psycinfo&t:ac=RecentSearches) \| \| Select item 1 \| **S1** \| [Sedentary OR Sedentary Behaviour OR Sedentary Behavior OR Sedentary lifestyle Or Sedentary time OR Sedentariness OR Physical inactivity Or Physically inactive OR Physical* Inactivit* OR Underactive Or Under Active OR low energy expenditure OR <1.5 MET OR Prolonged Sitting OR Sit* OR Reclin* OR Seated OR Stationary OR Sitting OR Sedentary Time OR Sedentary Bouts OR Sedentary Breaks](https://search.proquest.com/recentsearches.recentsearchtabview.recentsearchesgridview.scrolledrecentsearchlist.checkdbssearchlink:rerunsearch/3E5824B1BD554FA1PQ/None?site=psycinfo&t:ac=RecentSearches) \| [**164,840**](https://search.proquest.com/recentsearches.recentsearchtabview.recentsearchesgridview.scrolledrecentsearchlist.checkdbssearchlink_0:rerunsearch/3E5824B1BD554FA1PQ/None?site=psycinfo&t:ac=RecentSearches) \| |
| **6 SportDiscus Search** S6  (TI Adult OR Adults OR Men OR Women OR Individuals OR Employees OR Patients OR Seniors OR Elderly NOT (Child OR Children OR Infant OR Adolescent OR Teenager OR Teen) OR AB Adult OR Adults OR Men OR Women OR Individuals OR Employees OR Patients OR Seniors OR Elderly NOT (Child OR Children OR Infant OR Adolescent OR Teenager OR Teen) OR KW Adult OR Adults OR Men OR Women OR Individuals OR Employees OR Patients OR Seniors OR Elderly NOT (Child OR Children OR Infant OR Adolescent OR Teenager OR Teen [...](javascript:showHistoryTerm('ctl00_ctl00_MainContentArea_MainContentArea_historyControl_HistoryRepeater_ctl06_ellipsis',true))  **Limiters** - Published Date: 20000101-20201231  **Expanders** - Apply equivalent subjects  **Narrow by Language:**- english  **Search modes** - Boolean/Phrase [**View Results**](javascript:__doPostBack('ctl00$ctl00$MainContentArea$MainContentArea$historyControl$HistoryRepeater$ctl06$linkResults','')) (130)  S5  (TI Adult OR Adults OR Men OR Women OR Individuals OR Employees OR Patients OR Seniors OR Elderly NOT (Child OR Children OR Infant OR Adolescent OR Teenager OR Teen) OR AB Adult OR Adults OR Men OR Women OR Individuals OR Employees OR Patients OR Seniors OR Elderly NOT (Child OR Children OR Infant OR Adolescent OR Teenager OR Teen) OR KW Adult OR Adults OR Men OR Women OR Individuals OR Employees OR Patients OR Seniors OR Elderly NOT (Child OR Children OR Infant OR Adolescent OR Teenager OR Teen [...](javascript:showHistoryTerm('ctl00_ctl00_MainContentArea_MainContentArea_historyControl_HistoryRepeater_ctl07_ellipsis',true))  **Limiters** - Published Date: 20000101-20201231  **Expanders** - Apply equivalent subjects  **Search modes** - Boolean/Phrase [**View Results**](javascript:__doPostBack('ctl00$ctl00$MainContentArea$MainContentArea$historyControl$HistoryRepeater$ctl07$linkResults','')) (130)  S4  (TI Adult OR Adults OR Men OR Women OR Individuals OR Employees OR Patients OR Seniors OR Elderly NOT (Child OR Children OR Infant OR Adolescent OR Teenager OR Teen) OR AB Adult OR Adults OR Men OR Women OR Individuals OR Employees OR Patients OR Seniors OR Elderly NOT (Child OR Children OR Infant OR Adolescent OR Teenager OR Teen) OR KW Adult OR Adults OR Men OR Women OR Individuals OR Employees OR Patients OR Seniors OR Elderly NOT (Child OR Children OR Infant OR Adolescent OR Teenager OR Teen [...](javascript:showHistoryTerm('ctl00_ctl00_MainContentArea_MainContentArea_historyControl_HistoryRepeater_ctl08_ellipsis',true))  **Expanders** - Apply equivalent subjects  **Search modes** - Boolean/Phrase [**View Results**](javascript:__doPostBack('ctl00$ctl00$MainContentArea$MainContentArea$historyControl$HistoryRepeater$ctl08$linkResults','')) (132)  S3  TI ( Adult OR Adults OR Men OR Women OR Individuals OR Employees OR Patients OR Seniors OR Elderly NOT (Child OR Children OR Infant OR Adolescent OR Teenager OR Teen) ) OR AB ( Adult OR Adults OR Men OR Women OR Individuals OR Employees OR Patients OR Seniors OR Elderly NOT (Child OR Children OR Infant OR Adolescent OR Teenager OR Teen) ) OR KW ( Adult OR Adults OR Men OR Women OR Individuals OR Employees OR Patients OR Seniors OR Elderly NOT (Child OR Children OR Infant OR Adolescent OR Teenage [...](javascript:showHistoryTerm('ctl00_ctl00_MainContentArea_MainContentArea_historyControl_HistoryRepeater_ctl09_ellipsis',true))  **Expanders** - Apply equivalent subjects  **Search modes** - Boolean/Phrase [**View Results**](javascript:__doPostBack('ctl00$ctl00$MainContentArea$MainContentArea$historyControl$HistoryRepeater$ctl09$linkResults','')) (406,619)  S2  TI ( Randomised Controlled trial OR Randomized controlled Trial OR Randomised Control trial OR Randomized control Trial OR Randomised Clinical trial OR randomized clinical trial OR Randomised trial OR Randomized trial OR Controlled clinical trial OR Controlled Trial OR control trial OR RCT OR RCTS or RCT’s ) OR AB ( Randomised Controlled trial OR Randomized controlled Trial OR Randomised Control trial OR Randomized control Trial OR Randomised Clinical trial OR randomized clinical trial OR Random [...](javascript:showHistoryTerm('ctl00_ctl00_MainContentArea_MainContentArea_historyControl_HistoryRepeater_ctl10_ellipsis',true))  **Expanders** - Apply equivalent subjects  **Search modes** - Boolean/Phrase [**View Results**](javascript:__doPostBack('ctl00$ctl00$MainContentArea$MainContentArea$historyControl$HistoryRepeater$ctl10$linkResults','')) (20,300)  S1  TI ( Sedentary Behaviour OR Sedentary Behavior OR Sedentary lifestyle Or Sedentary time OR Sedentariness OR Physical inactivity Or Physically inactive OR Physically Underactive Or Underactive lifestyle OR low energy expenditure OR <1.5 MET OR Prolonged Sitting OR prolonged reclining OR Sitting time OR Sedentary Bouts OR Sedentary Breaks ) OR AB ( Sedentary Behaviour OR Sedentary Behavior OR Sedentary lifestyle Or Sedentary time OR Sedentariness OR Physical inactivity Or Physically inactive OR Ph [...](javascript:showHistoryTerm('ctl00_ctl00_MainContentArea_MainContentArea_historyControl_HistoryRepeater_ctl11_ellipsis',true))  **Expanders** - Apply equivalent subjects  **Search modes** - Boolean/Phrase [**View Results**](javascript:__doPostBack('ctl00$ctl00$MainContentArea$MainContentArea$historyControl$HistoryRepeater$ctl11$linkResults','')) (5,968) |
| **Grey literature search** ClinicalTrials.gov. Websites of organisations that report sedentary behaviour research, such as the Sedentary Behaviour Research Network, the World Health Organisation and the US Centers for Disease Control and Prevention |
