# Supplementary material for: Efficacy, characteristics, behavioural models and behaviour change strategies, of non-workplace interventions specifically targeting sedentary behaviour; a systematic review and meta-analysis of randomised control trials in healthy ambulatory adults
Source: PLoS One. 2021 Sep 7;16(9):e0256828. doi: 10.1371/journal.pone.0256828 (PMC8423252; doi:10.1371/journal.pone.0256828)
Supplement: S2 Table — Full-text articles excluded (n = 38) reasons; workplace (n = 18); SB not sole primary target of intervention (n = 8); older adult (n = 1); workplace and SB not sole primary target of intervention (n = 5); workplace and not randomised (n = 1); workplace and SB not sole primary target of intervention and not randomised (n = 2); SB not sole primary target of intervention and older adults (n = 3);. (DOCX) [file pone.0256828.s002.docx]

**S2 Table Studies excluded and reasons**

|  | **Studies excluded at full text screen** | **Title** | **Reason for exclusion** |
| --- | --- | --- | --- |
|  | Barwais, 2013 | Physical activity, sedentary behavior and total wellness changes among sedentary adults: a 4-week randomized controlled trial | SB not sole primary outcome |
|  | Bickmore, 2013 | A randomized controlled trial of an automated exercise coach for older adults | SB not primary outcome & Older adults |
|  | Blake, 2019 | Move-It: A Cluster-Randomised Digital Worksite Exercise Intervention in China: Outcome and Process Evaluation | Workplace |
|  | Carr, 2013 | Multicomponent intervention to reduce daily sedentary time: A randomised controlled trial | SB not sole primary outcome & Workplace |
|  | Chau, 2014 | The effectiveness of sit-stand workstations for changing office workers’ sitting time: results from the Stand@Work randomized controlled trial pilot | Workplace |
|  | Chau, 2016 | More standing and just as productive: effects of a sit-stand desk intervention on call center workers' sitting, standing, and productivity at work in the Opt to Stand pilot study | Not randomised &  Workplace |
|  | Chiang, 2019 | Motivational Counseling to Reduce Sedentary Behaviors and Depressive Symptoms and Improve Health-Related Quality of Life Among Women With Metabolic Syndrome | SB not sole primary outcome |
|  | Danquah, 2017 | Take a Stand!-a multi-component intervention aimed at reducing sitting time among office workers-a cluster randomized trial | Workplace |
|  | De Cocker, 2016 | The Effectiveness of a Web-Based Computer-Tailored Intervention on Workplace Sitting: A Randomized Controlled Trial | Workplace |
|  | De Jong, 2018 | Breaking up Sedentary Time in Overweight/Obese Adults on Work Days and Non-Work Days: results from a Feasibility Study | SB not sole primary outcome |
|  | Dutta, 2014 | Using sit-stand workstations to decrease sedentary time in office workers: a randomized crossover trial | Workplace |
|  | Edwardson, 2018 | Effectiveness of the Stand More AT (SMArT) Work intervention: cluster randomised controlled trial | Workplace |
|  | Fanning, 2016 | Effects of a DVD-delivered exercise program on patterns of sedentary behavior in older adults: a randomized controlled trial | SB not primary outcome (secondary analysis of prior data)  Older adults |
|  | Graves, 2015 | Evaluation of sit-stand workstations in an office setting: a randomised controlled trial | Workplace |
|  | Gao, 2016 | Effects of environmental intervention on sedentary time, musculoskeletal comfort and work ability in office workers | SB not sole primary outcome  Not randomised  Workplace |
|  | Garrett, 2019 | Computer-based Prompt's impact on postural variability and sit-stand desk usage behavior; a cluster randomized control trial | SB not sole primary outcome & Workplace |
|  | Gilson, 2009 | Do walking strategies to increase physical activity reduce reported sitting in workplaces: A randomized control trial | SB not primary outcome (PA) & Workplace |
|  | Healy, 2016 | A Cluster Randomized Controlled Trial to Reduce Office Workers' Sitting Time: Effect on Activity Outcomes | Workplace |
|  | Lakerveld, 2013 | The effects of a lifestyle intervention on leisure-time sedentary behaviors in adults at risk: the Hoorn Prevention Study, a randomized controlled trial | SB not primary outcome (healthy lifestyle) |
|  | Li, 2017 | Reducing Office Workers' Sitting Time at Work Using Sit-Stand Protocols: results From a Pilot Randomized Controlled Trial | Workplace |
|  | MacEwen, 2017 | Sit-stand desks to reduce workplace sitting time in office workers with abdominal obesity: A randomized controlled trial | Workplace |
|  | Mackey, 2015 | Do ergonomic and education interventions reduce prolonged occupational sitting? a randomised controlled trial | Workplace |
|  | Mantzari, 2019 | Impact of sit-stand desks at work on energy expenditure, sitting time and cardio-metabolic risk factors: Multiphase feasibility study with randomised controlled component | Workplace & SB not Primary outcome |
|  | Maylor, 2018 | Efficacy of a Multicomponent Intervention to Reduce Workplace Sitting Time in Office Workers: A Cluster Randomized Controlled Trial | Workplace |
|  | Neuhaus, 2014 | Workplace sitting and height-adjustable workstations: A randomized controlled trial | Workplace |
|  | Otten, 2009 | Effects of Television Viewing Reduction on Energy Intake and Expenditure in Overweight and Obese Adults: A Randomized Controlled Trial | SB not Primary outcome |
|  | Parry, 2013 | Participatory workplace interventions can reduce sedentary time for office workers--a randomised controlled trial | Workplace |
|  | Puig-Ribera, 2015 | Patterns of impact resulting from a 'sit less, move more' web-based program in sedentary office employees | Workplace |
|  | Rosenberg, 2020 | Reducing Sitting Time in Obese Older Adults: The I-STAND Randomized Controlled Trial | Older adult |
|  | Santos, 2019 | Effects of High-Intensity Interval and Moderate-Intensity Continuous Exercise on Physical Activity and Sedentary Behavior Levels in Inactive Obese Males: a Crossover Trial | SB + PA (combined outcomes) |
|  | Schuna, 2014 | Evaluation of a workplace treadmill desk intervention: A randomized controlled trial | Workplace |
|  | Stephens, 2014 | Intervening to reduce workplace sitting time: how and when do changes to sitting time occur? | Not randomised  SB not primary outcome &  Workplace |
|  | Taylor, 2016 | Impact of Booster Breaks and Computer Prompts on Physical Activity and Sedentary Behavior Among Desk-Based Workers: A Cluster-Randomized Controlled Trial | SB + PA (combined outcomes)  & workplace |
|  | Tucker, 2016 | Worksite Physical Activity Intervention for Ambulatory Clinic Nursing Staff | Workplace |
|  | Verweij, 2012 | The application of an occupational health guideline reduces sedentary behaviour and increases fruit intake at work: results from an RCT | SB not sole primary outcome |
|  | Weatherson, 2020 | Impact of a low-cost standing desk on reducing workplace sitting (StandUP UBC): a randomised controlled trial | Workplace |
|  | White, 2017 | On Your Feet to Earn Your Seat: pilot RCT of a theory-based sedentary behaviour reduction intervention for older adults | SB + PA (combined outcomes) & Older adults |
|  | Wyke, 2019 | The effect of a programme to improve men’s sedentary time and physical activity: The european fans in training (EuroFIT) randomised controlled trial | SB + PA (combined outcomes) |

Full-text articles excluded (n = 38) reasons;

workplace (n = 18); SB not sole primary target of intervention (n = 8); older adult (n = 1);

workplace *and* SB not sole primary target of intervention (n = 5);

workplace *and* not randomised (n = 1);

workplace *and* SB not sole primary target of intervention *and* not randomised (n=2);

SB not sole primary target of intervention *and* older adults (n=3);
