# Supplementary material for: Efficacy, characteristics, behavioural models and behaviour change strategies, of non-workplace interventions specifically targeting sedentary behaviour; a systematic review and meta-analysis of randomised control trials in healthy ambulatory adults
Source: PLoS One. 2021 Sep 7;16(9):e0256828. doi: 10.1371/journal.pone.0256828 (PMC8423252; doi:10.1371/journal.pone.0256828)
Supplement: S3 Table — Note; Only Biddle reported any follow up measures; SF = self feedback; TF = tailored feedback. (DOCX) [file pone.0256828.s003.docx]

S3 Table Anthropometric Measures and Biomarkers

Note; Only Biddle reported any follow up measures; SF= self feedback; TF= tailored feedback.
