# Supplementary material for: Efficacy, characteristics, behavioural models and behaviour change strategies, of non-workplace interventions specifically targeting sedentary behaviour; a systematic review and meta-analysis of randomised control trials in healthy ambulatory adults
Source: PLoS One. 2021 Sep 7;16(9):e0256828. doi: 10.1371/journal.pone.0256828 (PMC8423252; doi:10.1371/journal.pone.0256828)
Supplement: S4 Table — (DOCX) [file pone.0256828.s004.docx]

S3 Table activPAL weartime protocol and criteria for inclusion in analysis

| Study | activPAL weartime protocol | | | inclusion in analysis criteria | |
| --- | --- | --- | --- | --- | --- |
|  | Number of days | Hours / day | Daily log | Minimum  number days | Minimum  hours /day |
| Aadahl  2014 | 7 | 24 (except bathing) | Yes | 2 | >22 |
| Arrogi  2019 | 14 | waking hours  (except bathing) | Yes | 3 weekday &  1 weekend day | 12 |
| Biddle  2015 | 10 | 24  (waterproof dressing) | Not reported | 4 | 10  (movement data) |
| Ellingson  2016 | 7 | waking hours  (except bathing) | Yes | 4 including  1 weekend day | 10 |
| Judice  2015 | 14 | 24 (except bathing) | Yes | Not reported | >22 |
| Kittigawa  2020 | 7 | 24 (except bathing) | Not reported | Not reported | Not reported |
| Nishimuru  2019 | 7 | 9 (from 9am-6pm) | Not reported | 2 weekdays &  1 weekend day | 8 |
